# Proteomic and functional profiling of suspended jejunal enteroids reveals pharmacokinetic fidelity to native intestine

**DOI:** 10.1016/j.isci.2026.117654

**Published:** 2026-09-30

**Authors:** Foteini Tzioufa, Merve Ceylan, Maria Letizia Di Martino, Dinh Son Vo, Ana C.C. Lopes, Rebekkah Hammar, Magnus Sundbom, Martin Skogar, Per M. Hellström, Dominic-Luc Webb, Maria Karlgren, Iain Gardner, Patrik Lundquist, Jens Eriksson, Daisy Hjelmqvist, Mikael E. Sellin, Madlen Hubert, Per Artursson

**Affiliations:** 1 Department of Pharmacy, Uppsala University, 752 37 Uppsala, Sweden; 2 Department of Medical Biochemistry and Microbiology, Uppsala University, 752 37 Uppsala, Sweden; 3 Certara UK Ltd, Simcyp Division, Level 2-Acero, Sheffield S1 2BJ, UK; 4 Department of Surgical Sciences, Uppsala University, 751 85 Uppsala, Sweden; 5 Department of Medical Sciences, Uppsala University, 751 85 Uppsala, Sweden; 6 DLW Bioanalytics, 195 33 Märsta, Sweden; 7 Science for Life Laboratory, 752 37 Uppsala, Sweden

**Keywords:** human small intestine, enteroids, proteomics, live-cell microscopy, drug transport, drug metabolism, PBPK modeling

## Abstract

Intestinal enteroids are “mini-guts” that recapitulate essential features of the intestinal epithelium *in vitro*. We developed and characterized native basal-out (BO) and morphogenically everted apical-out (AO) jejunal enteroids cultured in suspension. Using quantitative proteomics and a range of advanced microscopy techniques, we show that these enteroids progressively adopt protein profiles closely resembling those of freshly isolated villus enterocytes and human jejunal tissue. Optimally differentiated enteroids exhibit mature epithelial morphology, intact barrier function, nutrient absorption, and clinically relevant transporter activity (P-glycoprotein; Pgp/*ABCB1*, breast cancer resistance protein; BCRP/*ABCG2*), reflecting *in vivo* physiology. Metabolic assessment of orally administered cytochrome P450 3A4 (CYP3A4) probe drugs, terfenadine and midazolam, accurately predicted first-pass metabolism and clinical pharmacokinetics via physiologically based pharmacokinetic (PBPK) modeling. These findings establish suspension-grown 3D jejunal enteroids as a translationally relevant, human-derived platform that is advantageous over conventional cell line and animal models for studying drug disposition, nutrient transport, and intestinal barrier function.

## Highlights

• Human jejunal enteroids closely mimic mature villus enterocytes
• Apical-out enteroids enable direct studies of epithelial transport
• Enteroids reproduce human CYP3A4 metabolism and drug transport
• PBPK modeling predicts clinical pharmacokinetics from enteroid data

## Graphical abstract

**Figure:**
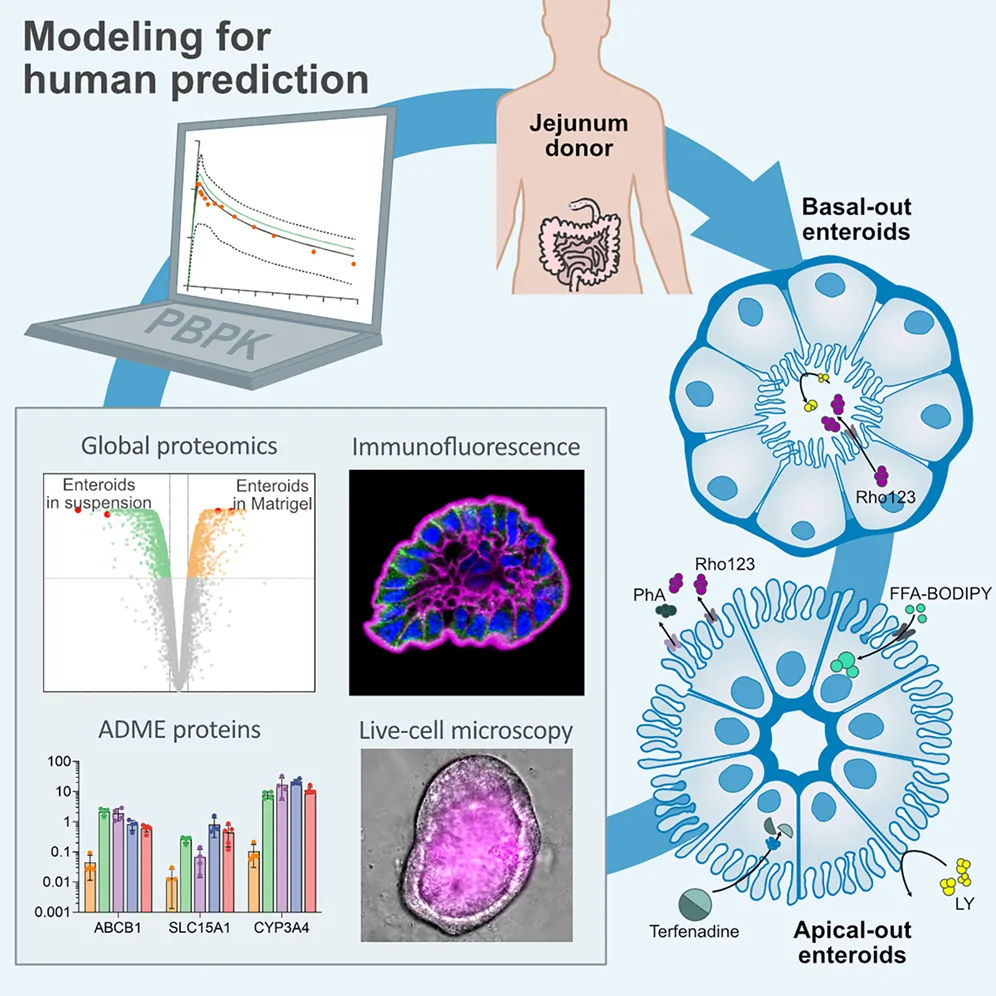

## Teaser

Bioengineering; Biotechnology; Cell biology; Omics

## Introduction

The single layer of epithelial cells lining the inner lumen of the human intestine provides a barrier against ingested noxious compounds and microorganisms. It simultaneously assures absorption of nutrients and other essential food constituents such as vitamins and trace elements. Most nutrients are absorbed by mature absorptive enterocytes at the villus tips in the upper part of the small intestine, the jejunum.^1^ This is also the major absorption site for orally administered drugs. It is thus not surprising that the selective barrier function of the jejunal epithelium is investigated in drug discovery and development.^2^ Though *in vitro* models of the human jejunum are of great interest, thus far no model has encompassed all the features of *in vivo* absorptive villus enterocytes.

Absorptive enterocytes originate from crypt stem cells and differentiate during their journey along the crypt-villus axis into fully differentiated enterocytes at the tips of the villi. By this point, enterocytes have developed the features required to perform their essential dual barrier and absorptive functions for a short period before they are shed off and are replaced by new cells. The lifespan of human enterocytes is 3–5 days,^3^ making them notoriously difficult to maintain in cell culture for functional studies. Freshly isolated villus cells in suspension maintain *in vivo*-like features such as metabolic capacity for a few hours, but their functions and viability decline after isolation.^4^^,^^5^ In the absence of primary cell models, the colorectal adenocarcinoma-derived Caco-2 cell line has been used in mechanistic studies ranging from transport mechanisms of essential food constituents and drugs to barrier dynamics, e.g., after exposure to enteropathogens.^6^^,^^7^ Caco-2 cells have a phenotype of villus enterocytes, form a tight junction barrier, and express functional nutrient transporters. However, due to their colorectal adenocarcinoma origin, Caco-2 cells also have abnormalities, including an aberrant panel of metabolic enzymes and truncated glycosylation patterns compared with the native intestinal epithelium.^8^^,^^9^ Thus, their barrier to infectious microorganisms and nanoparticles is reduced compared to *in vivo* tissue.^10^^,^^11^

Organoids of different origins represent a major advance throughout the entire field of biomedicine.^12^^,^^13^^,^^14^ Organoids from adult human tissue stem cells are now an important tool for studies of normal and pathological conditions of the mucosal barrier. Intestinal epithelial organoids originating from adult stem cells, termed enteroids if from the small intestine, preserve important functions of the intestinal epithelium^15^ and can be grown in different configurations. These include two-dimensional (2D) monolayers,^16^ three-dimensional (3D) enteroids entrapped in an extracellular matrix as originally described,^17^^,^^18^ and 3D enteroid suspensions,^19^^,^^20^ each with their advantages and disadvantages. A drawback with the original 3D basal-out (BO) configuration is that the apical surface is facing an inner confined lumen that can only be accessed by perforation of the epithelial barrier, e.g., by microinjection. This complication can be addressed by dispersing the enteroids and reseeding them as 2D monolayers on permeable supports.^21^ The 2D configuration has been used to study nutrient and drug transport, metabolic function,^22^^,^^23^^,^^24^ as well as microbial infection.^25^ An improved protocol for expansion and differentiation of jejunal enteroids into 2D monolayers was recently described by some of us. These 2D monolayers had barrier features of the human absorptive villus cells and provided improved protection against *Salmonella* infection,^25^ but in contrast to the observations by others^26^ many membrane transporters and metabolizing enzymes still had markedly lower expression levels than observed for native epithelial tissue. The intestinal epithelium senses curvature, exemplified by crypt-like regions of enteroids preferentially localizing in indented areas of a supporting scaffold, while differentiated villus cells cover the finger-like projections.^27^

We reasoned that a curvature mimicking the latter would promote the development of fully differentiated absorptive enterocytes. Morphogenically everted 3D apical-out (AO) enteroids in suspension, as originally described by Co et al.*,*^19^ recapitulate the convex curvature of villus tips. We, therefore, set out to characterize and investigate these enteroids for functional studies of nutrient and, in particular, drug transport and metabolism, using primary jejunal enterocytes and tissue as proteomic references. By monitoring relevant markers via quantitative global proteomics, optimized growth, and differentiation conditions for human jejunal enteroids were identified. We then compared the results with those of BO enteroids, jejunal enterocytes, and tissues, partly isolated from the same individuals. Live-cell microscopy techniques were developed to characterize barrier and transport functions in the enteroid population. Appropriate function was demonstrated for active fatty acid uptake as well as active efflux via two clinically relevant ABC transporters: P-glycoprotein (Pgp/*ABCB1*) and breast cancer resistance protein (BCRP/*ABCG2*). Finally, we investigated the metabolism of two orally administered probe drugs (terfenadine and midazolam) for the major intestinal drug-metabolizing enzyme cytochrome P450 3A4 (CYP3A4), normalized to enzyme expression, and predicted the clinical pharmacokinetics of each of the drugs using physiologically based pharmacokinetic (PBPK) models. Our results demonstrated that properly differentiated AO enteroids closely resemble fully mature absorptive villus enterocytes, both morphologically and at the proteomic level, with expression of many major functional proteins at levels comparable to those in human jejunal tissue. Together, these features establish suspension-grown 3D jejunal enteroids as a robust and translationally relevant human model for studying intestinal epithelial function and advancing preclinical research in drug absorption, metabolism, and barrier integrity.

## Results

### Establishment of optimally differentiated enteroids from human jejunal tissue

We first established conditions for the growth and differentiation of three types of 3D enteroids: Matrigel-embedded basal-out cultures, BO suspension cultures, and morphogenically everted AO orientation (Figure 1A). We benchmarked these cultures against freshly isolated enterocytes and jejunal villus tissue. We found that a previously optimized protocol for 2D cultures,^25^ in which the enteroids were cultivated for 4–6 days, best suited our purposes (Figure 1B). Less than four days of culture in Organoid Differentiation Medium (ODM) resulted in less differentiated cells, while longer incubation periods led to a gradual increase in cell death, likely caused by terminally differentiated cells.

**Figure 1:**
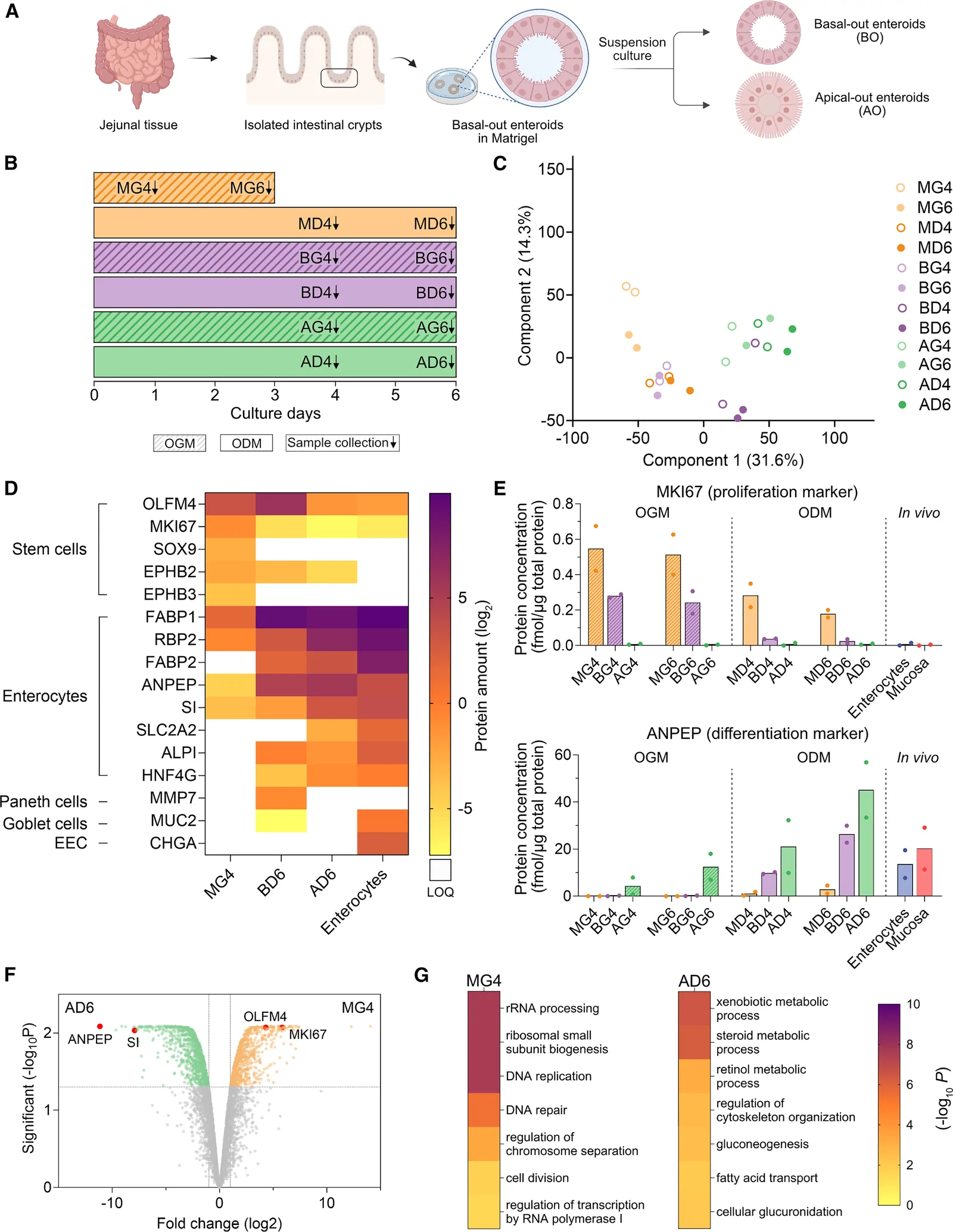
Global protein profiling of human adult stem cell-derived enteroids in different culture formats (A) Schematic of enteroid generation from human tissue samples, followed by culture in Matrigel and suspension formats. (B) Schematic of the differentiation protocol. 3D enteroids were initially expanded in Matrigel with OGM for 3 days (not shown on the timeline for simplicity). Enteroids were then cultured in various formats: 3D Matrigel enteroids were either maintained in OGM (MG4, MG6) or differentiated in ODM for 4–6 days (MD4, MD6). Basal-out enteroids in suspension were cultured for 4–6 days in either OGM (BG4, BG6) or ODM (BD4, BD6) with 7.5% Matrigel. Apical-out enteroids in suspension were also cultured in OGM (AG4, AG6) or ODM (AD4, AD6) for 4–6 days. (C) Principal component analysis (PCA) of protein expression profiles across all enteroid samples, highlighting variations between different culture formats. (D) Proteome profiles of 3D basal-out and apical-out enteroids after 6 days in suspension culture. Matrigel-grown enteroids in OGM (MG4) served as a stemlike control, while primary jejunal enterocytes represented differentiated villus enterocytes. LOQ indicates expression levels below the limit of quantification. (E) Quantification of MKI67 (proliferation marker, top) and aminopeptidase N (differentiation marker, bottom) across 3D enteroid formats (M: Matrigel, B: basal-out, A: apical-out) cultured in OGM or ODM for 4–6 days, compared to primary enterocyte and jejunal mucosa *in vivo* levels. Data points refer to technical replicates of one donor (enteroids) or two donors (enterocytes, jejunal mucosa), and the bar indicates the mean. (F) Volcano plot of differentially expressed proteins between Matrigel-grown enteroids in OGM (day 4, MG4, orange) and apical-out enteroids in ODM (day 6, AD6, green), with corresponding enrichment analysis. Orange and green dots represent proteins significantly upregulated or downregulated (adjusted *p* value ≤ 0.05, fold change ≥2) in MG4 (789 proteins) compared to AD6 (1014 proteins). (G) Heatmap of GO enrichment of biological processes in MG4 and AD6. (C–G) Data from different donors are shown (optimal enteroid culture conditions were characterized using donor Hu18-9jej; enterocyte and jejunal mucosa donors Hu21-7jej and Hu21-14jej). See also Figure S1 and Table S1.

The differentiation into villus-like enteroids was followed by global proteomics (Figures 1C–1G). Principal component analysis (PCA) of the proteomes revealed clear, time-dependent separation of Matrigel-grown enteroids (“MG” and “MD”), from basal-out (“BG” and “BD”) to apical-out enteroid (“AG” and “AD”) suspensions (Table 1; Figure 1C). As expected, MG4 enteroids, maintained in growth promoting medium (Organoid Growth Medium; OGM), retained proliferative capacity (increased marker of proliferation Ki-67; MKI67) and expressed markers of an immature phenotype (e.g., OLFM4, SOX9, EPHB3) while most enterocyte markers were present at moderate or low levels (Figure 1D). In contrast, by day 6, AO enteroids (AD6), maintained in ODM, the differentiation promoting medium, expressed several proliferation and stem cell markers below their detection levels, while most markers for absorptive enterocytes, such as brush border enzymes, were increased compared to jejunal tissue levels (Figures 1D and S1). The expression of markers for goblet, enteroendocrine, and Paneth cells was either below or close to the limit of quantification, highlighting the dominance of the absorptive cell lineage. BO enteroids at day 6 (BD6) showed an intermediate phenotype compared to MG4 and AD6, with low expression of stem cell markers and increased expression of absorptive enterocyte markers. The gradual maturation toward a more differentiated phenotype is exemplified for all growth conditions by comparison to freshly isolated enterocytes and jejunal villus tissue, using the proliferation marker MKI67 and the brush border enzyme Aminopeptidase N (ANPEP) as a differentiation marker (Figure 1E). Consistent patterns across culture conditions were also observed for other markers, such as CD44 (proliferation marker) and SI (absorptive cell marker, Figure S1).

**Table 1:** Nomenclature of enteroids in different culture conditions presented in Figure 1

	Enteroids in Organoid Growth Medium (OGM)		Enteroids in Organoid Differentiation Medium (ODM)	
	Day 4	Day 6	Day 4	Day 6
Basal-out in matrigel	MG4	MG6	MD4	MD6
Basal-out in suspension	BG4	BG6	BD4	BD6
Apical-out in suspension	AG4	AG6	AD4	AD6

Of the 7037 quantified proteins, differential expression analysis identified 1803 proteins distinguishing MG4 and AD6, enriched for proliferation versus xenobiotic metabolism and epithelial differentiation, respectively (Figures 1E–1G). Overall, AD6 enteroids most closely resembled jejunal enterocytes, while BD6 represented an intermediate but differentiated state. We therefore mainly focused our subsequent studies on AD6 and BD6 enteroids in suspension. However, we included the MD6 proteomes for comparison. For the remainder of the study, we simplify the nomenclature and refer to MD6, BD6 and AD6 as MBO, BO and AO, respectively.

### Apical-out and basal-out enteroids provide an intact epithelial barrier

The 3D AO and BO enteroids had a continuous monolayer of enterocytes (Figure 2). The AO enteroids were almost spherical (circularity around 0.9), with an average diameter of approximately 140 μm (Figure 2C; Table S2). Cell shape was comparable to that of human enterocytes (polygonal cells with a height of 30 μm and width of 11 μm).^28^ This resulted in an average number of slightly more than 700 ± 100 enterocytes per enteroid (Figures 2A, 2C, and 2D; Table S2). Scanning electron microscopy (SEM) showed that the polygonal cell surfaces were covered by a dense carpet of microvilli, characteristic of a mature villus enterocyte phenotype (Figure 2A). Occasionally, AO enteroids displayed ongoing cell extrusion (Figure S2A). In intestinal tissue, such extruded enterocytes appear healthy, although a subset is caspase-3 (CASP3)-positive.^29^ Apoptosis markers in differentiated enteroids were confirmed by early (CASP8, elevated vs. tissue, Figure S2B) and late markers (CASP3, CASP7 slightly lower vs. tissue, Figures S2C and S2D).

**Figure 2:**
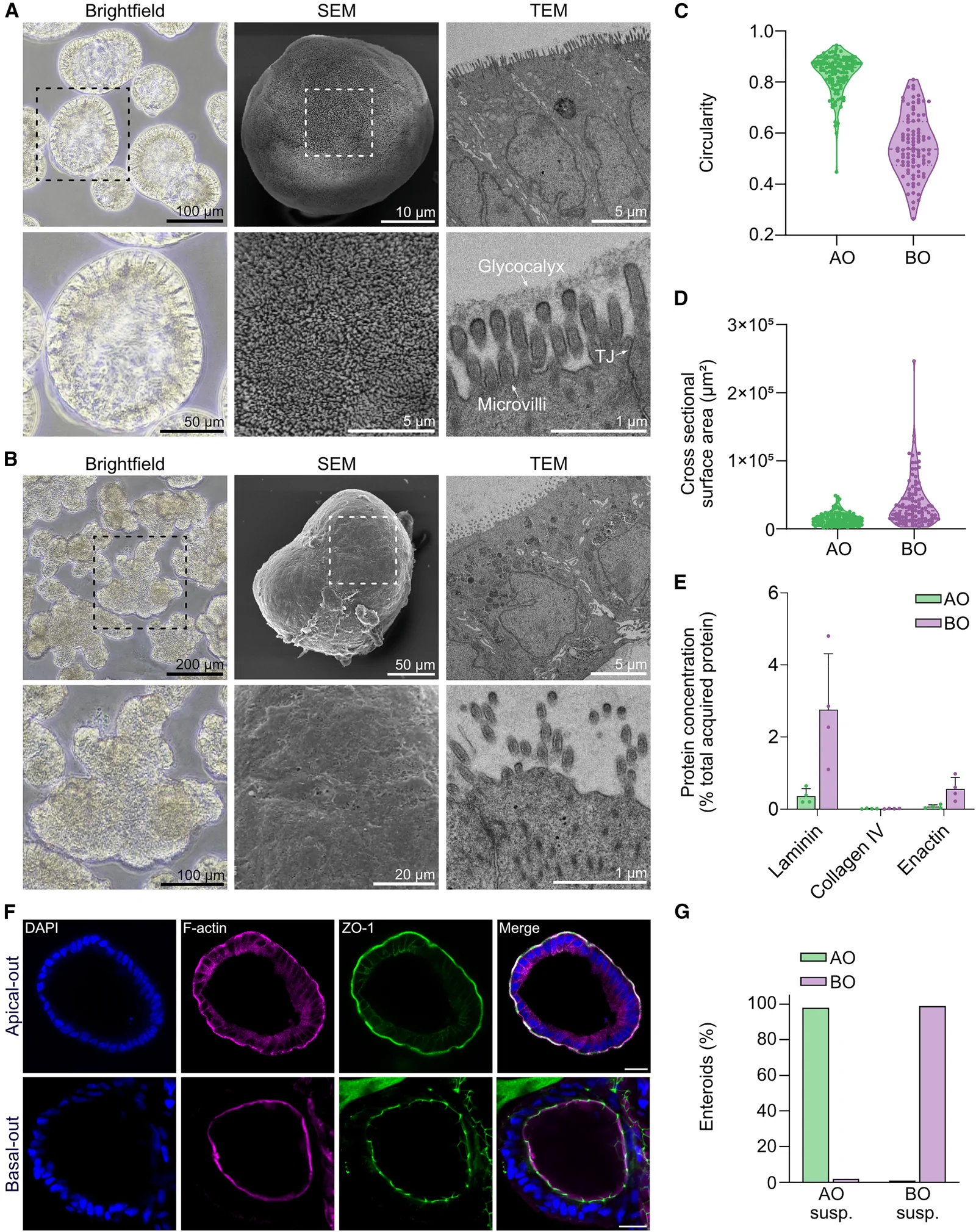
Characterization of morphology, size, and shape distribution of differentiated 3D enteroids in suspension (A) Representative micrographs of AO enteroids: bright-field microscopy shows live enteroids in suspension (left), scanning electron microscopy (SEM) reveals a dense microvillar surface (middle), and transmission electron microscopy (TEM) shows the organized microvilli-rich brush border, tight junctions (TJ), and glycocalyx (right). High-magnification brightfield and SEM images (bottom row) correspond to the areas outlined by dotted squares in the upper row. (B) Representative micrographs of BO enteroids: live bright-field microscopy illustrates typical basal-out morphology (left), SEM shows the smooth basal surface (middle), and TEM reveals the microvilli-rich brush border on the lumen-facing apical surface, confirming basal-out polarity (right). High-magnification views (bottom row) correspond to the areas outlined in the upper row. (C) Circularity analysis of AO (*n* = 118) and BO enteroids (*n* = 94) in suspension from brightfield micrographs. (D) Total cross-sectional surface area at 6 days of suspension culture for AO (*n* = 122) and BO (*n* = 94) enteroids. (E) Concentrations of Matrigel proteins (% of protein over total acquired protein) present in AO and BO enteroids in suspension cultures after washing. Data from different donors (*n* = 4, Hu22-2jej, Hu22-3jej, Hu22-5jej, and Hu18-9jej) are shown as means +SD. (F) Representative confocal images following staining of zonula occludens-1 (ZO-1, green), phalloidin (F-actin; magenta) and nuclei (DAPI; blue), highlighting the continuous monolayer barrier in AO and BO enteroids. Scale bars, 20 μm. (G) Quantification of apical-out and basal-out polarity in enteroid suspensions by confocal microscopy (*n* = 100 per population). (A–D, F–G). Donor Hu18-9jej was used throughout. See also Figure S2–S4; Tables S2 and S3.

BO enteroids were larger and more heterogeneous than the AO enteroids, with an average diameter of approximately 230 μm and a circularity of about 0.5 (Figures 2B–2D; Table S2). The BO enteroids formed crypt-like protrusions as previously described.^30^^,^^31^^,^^32^ Unlike the morphologically homogeneous AO enteroids, the BO population included a subpopulation of smaller enteroids with a stem-like phenotype. These smaller structures displayed a more spherical shape and a thinner, less columnar cell layer, similar to what has been observed in fetal crypt-derived and induced pluripotent stem cell-derived BO enteroid suspension cultures (Figure S3).^27^ SEM showed a smooth basolateral surface without distinct cell borders, likely due to residual basement membrane components from Matrigel (Figure 2B). Notably, only small amounts of Matrigel remained after washing before imaging and subsequent experiments (Figures 2E and S4).

Transmission electron microscopy (TEM) confirmed a columnar morphology of both AO and BO enterocytes closely resembling villus enterocytes *in vivo*. These cells displayed basally located nuclei and well-developed tight and adherens junctions (Figures 2A and 2B). Proteins contributing to the normal appearance of microvilli^33^ were generally detected at concentrations comparable to *in vivo* levels and included: F-actin bundling and microvilli elongating proteins (e.g., VIL1, ESPN, EPS8), membrane crosslinking proteins (e.g., EZR, PDZK1, MYO1A), and intra-microvillar anchoring proteins (e.g., ANKS4B, CDHR2, CDHR5) (Table S3). The microvilli-anchored glycocalyx barrier was clearly visualized in the AO-oriented enteroids by TEM (Figure 2A), while it appeared diffuse in the BO, probably due to the presence of luminal deposits. Proteomic analysis further confirmed the presence of surface-bound mucins, including those integral to the glycocalyx structure (e.g., MUC1, MUC13, MUC17). Additionally, several fucosyltransferases (e.g., FUT2, FUT3, FUT8) were identified that mediate the fucosylation of surface mucins and other glycoproteins, fortifying the cell surface by creating complex branched carbohydrate structures (Table S3). In contrast, to the AO and BO enteroids, MBO showed substantially lower or no expression of several characteristic proteins of the microvillar barrier (e.g., MYO1A, ANKS4B, CDHR2, CDHR5, MUC13; Table S3).

Fluorescence staining showed that more than 98% of AO and BO enteroids had continuous actin belts and ZO-1 tight junctions, oriented toward the medium in AO and toward the lumen in BO enteroids, consistent with previous observations (Figures 2F and 2G).^34^^,^^35^ Proteomics confirmed that fundamental adherens (CDH1, CTNND1, VCL) and tight junction proteins (CLDNs, OCLN) were expressed at levels similar to those in jejunal tissue (Figure 3A; Table S4). Both gate (CLDN2, CLDN15) and fence claudins (CLDN3, CLDN7) were present.^35^ Desmosomal proteins, including extracellular adhesion proteins (e.g., DSG2 and DSC2) and intracellular anchoring proteins (e.g., DSP and PKP2), were expressed at levels comparable to those in tissues (Table S4). In MBO, the proteins of the junctional complex were generally expressed at levels similar to those in AO and BO.

**Figure 3:**
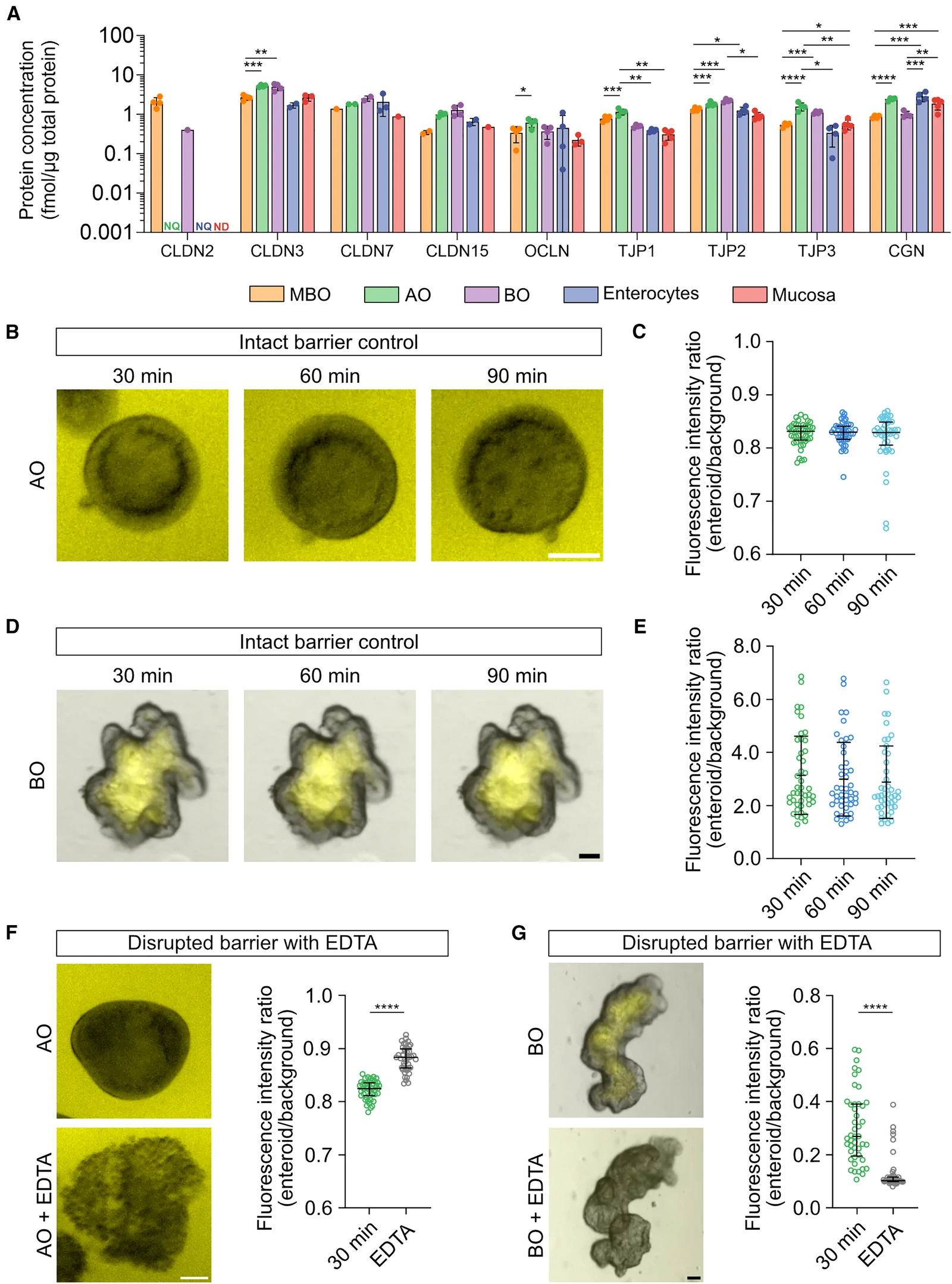
Epithelial barrier integrity assessment in 3D enteroids in suspension (A) Expression of important tight junction proteins in MBO, AO, and BO as compared to primary enterocyte and jejunal mucosa *in vivo* levels. Data are shown as mean ± SD. ∗*p* < 0.05, ∗∗*p* < 0.01, ∗∗∗*p* < 0.001, ∗∗∗∗*p* < 0.0001 (DEqMS); ND = not detected; NQ = not quantified. Data from different donors is shown (*n* = 4 enteroid donors Hu18-9jej, Hu22-2jej, Hu22-3jej, Hu22-5jej; *n* = 4 enterocyte donors Hu21-7jej, Hu21-14jej, Hu22-3jej, Hu22-5jej and *n* = 5 jejunal mucosa donors Hu21-7jej, Hu21-14jej, Hu22-2jej, Hu22-3jej, Hu22-5jej). (B and D) Representative time-lapse series showing suspended (B) AO and (D) BO enteroids that were incubated in medium containing lucifer yellow (LY, 100 μM) at 37°C to assess intact epithelial barrier over a time period of 90 min. AO enteroids were imaged live by confocal microscopy, and LY fluorescence intensity was determined within the outline of the enteroid and background. Scale bars, 50 μm. Images are representative of 44 AO and BO enteroid observations. (C and E) Quantification of LY fluorescence intensity within enteroids was normalized to background. For the experiment, an average of 44 enteroid observations per time point was made; the Kruskal-Wallis test showed no statistically significant difference between time points. Data are shown as median ± IQR. Data are representative as one of two independent experiments. (F and G) Incubation with Ca^2+^ chelator EDTA disrupts paracellular barrier integrity in (F) AO and (G) BO enteroids. The LY permeability assay was used to evaluate apical-out enteroids treated with EDTA for 30 min. Enteroids were imaged as described in (B and D) using live-cell confocal microscopy. Scale bars, 50 μm. The corresponding quantification of LY fluorescence intensity within untreated enteroids (incubation with LY for 30 min) and enteroids treated with EDTA (additional incubation for 30 min with EDTA) was normalized to background. Data are shown as median ± IQR. An average of 44 enteroid observations were analyzed; ∗∗∗∗*p* < 0.0001 (Mann-Whitney U test). (B–G) Enteroids from donor Hu18-9jej were used. See also Table S4.

We next investigated the epithelial barrier function of AO and BO enteroids, using lucifer yellow (LY; molecular weight 442.3 Da),^36^ a well-established low molecular weight hydrophilic marker for assessing epithelial barrier function.^37^ AO enteroids excluded LY for 90 min, confirming barrier integrity, while EDTA exposure, which disrupts calcium-dependent junctions, caused leakage (Figures 3B, 3C, and 3F). In contrast, BO enteroids accumulated LY within the lumen due to active efflux by the apical transporter BCRP (Figure 3D).^38^ To assess barrier function independently, BO enteroids were preloaded with LY; their luminal intensity remained stable for 90 min but declined drastically after EDTA exposure, demonstrating an intact paracellular barrier under normal conditions (Figures 3D, 3E, and 3G).

### Apical-out and basal-out enteroids express an *in vivo*-like panel of jejunal membrane transporters and metabolizing enzymes

The absorption of critical nutrients, vitamins, and trace elements in the human intestine is handled by membrane-spanning transport proteins, of which the solute carrier transporter (SLC) superfamily is the largest with more than 455 members arranged into 66 families.^39^ The three enteroid types (MBO, AO, BO) expressed 102–110 solute carrier (SLC) transporters, which is comparable to the 106–108 SLC transporters found in primary enterocytes and jejunal tissues (Table S5). Transporters for amino acids and small peptides (e.g., PEPT1/*SLC15A1*, EAAT3/*SLC1A1*, LAT1/*SLC7A5*), monosaccharides (e.g., SGLT1/*SLC5A1*, GLUT5/*SLC2A5*, GLUT2/*SLC2A2*) and fatty acids (e.g., *SLC27A1-4*), were often present at levels comparable to those in primary enterocytes and jejunal tissue (Figure 4; Figure S5; Table S6). Some essential transporters had a lower expression in the MBO enteroids (Figures 4B and 4C). Many other essential transporters were identified and quantified, including those transporting vitamins, small ions, and metabolites (Table S6).

**Figure 4:**
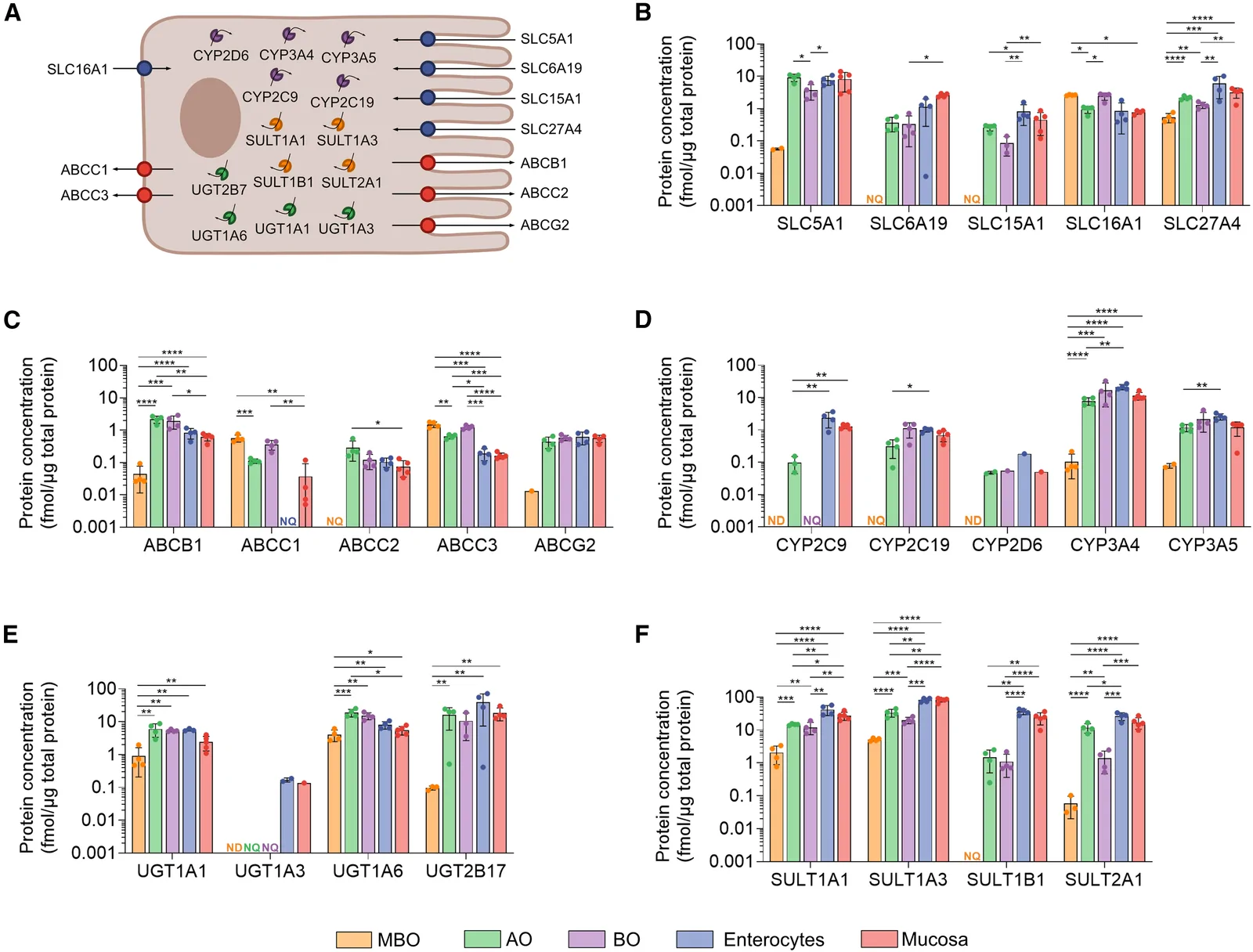
Protein expression levels of drug transporting and drug metabolizing enzymes in 3D enteroids (A) Schematic overview of important intestinal influx and efflux transporters, as well as phase I and II metabolizing enzymes. (B–F) Expression of key transporters such as (B) solute carrier (SLC) transporters and (C) ATP-binding cassette (ABC) transporters, as well as key enzymes for (D) cytochrome P450 (CYP) enzymes, (E) UDP-glucuronosyltransferases (UGT), (F) sulfotransferases (SULT), compared with primary enterocyte and jejunal mucosa *in vivo* levels. Data are shown as mean ± SD. ∗*p* < 0.05, ∗∗*p* < 0.01, ∗∗∗*p* < 0.001, ∗∗∗∗*p* < 0.0001 (DEqMS); ND = not detected; NQ = not quantified. Data from different donors are shown (*n* = 4 enteroid donors Hu18-9jej, Hu22-2jej, Hu22-3jej, Hu22-5jej; *n* = 4 enterocyte donors Hu21-7jej, Hu21-14jej, Hu22-3jej, Hu22-5jej and *n* = 5 jejunal mucosa donors Hu21-7jej, Hu21-14jej, Hu22-2jej, Hu22-3jej, Hu22-5jej) See also Tables S5, S6, and S7–S12.

The largest differences were observed for nutrient and metabolite transporters such as the glucose uptake transporter GLUT1/*SLC2A1* and MCT4/*SLC16A3*, which transports excess lactate and other monocarboxylates from the cell interior to the extracellular space.^40^^,^^41^ Both transporters were expressed at about 10-fold higher levels in the enteroids compared to primary enterocytes and jejunal tissue, likely reflecting enhanced glycolytic metabolism in cell culture, where increased glucose uptake drives lactate production that requires MCT4-mediated efflux to sustain metabolic flux^41^ (Table S6). Examples of nutrient transporters that had a lower expression in the enteroids than in jejunal tissue include B^0^AT1/*SLC6A19* and LAT2/*SLC7A8* (Table S6). This may reflect the absence of some physiological luminal, hormonal, and microbial cues required to maintain high expression.

We next investigated the expression of the ATP-binding cassette (ABC) transporter superfamily. (Table S7).^42^ Vital ABC-family members that are expressed in the plasma membrane of enterocytes function as efflux pumps that export metabolites and limit intestinal absorption of xenobiotics and toxins. As for the SLC transporters, the ABC transporter expression was often higher in AO and BO than in MBO enteroids (Figure 4C; Table S7). Another trend was that ABC transporters located in the membrane facing the nutritious cell culture medium tended to have a higher expression than those facing the closed inner luminal space. For AO-oriented enteroids, this trend is exemplified by the apically located ABC transporter MRP2/*ABCC2* (Figures 4A and 4C; Table S7). The corresponding examples for basolaterally located transporters in BO enteroids are MRP1/*ABCC1* and MRP3/*ABCC3* (Figures 4A and 4C; Table S7). Most ABC transporters were expressed at levels comparable to or higher than in jejunal tissue (Figure 4C; Table S7). One exception was the peroxisomal long-chain fatty acid transporter ALDP/*ABCD1*, which was 10- to 20-fold lower (Table S7). This is likely related to reduced peroxisomal fatty acid metabolism *in vitro* due to limited lipid exposure.

### Apical-out enteroids transport medium-chain free fatty acids via the FATP4 transporter

Next, we investigated the expression and function of the constitutive fatty acid transporter FATP4/*SLC27A4*. The expression of FATP4 was 3- and 6-fold lower in the AO and BO enteroids, respectively, compared to the primary enterocytes (Figure 4B). We confirmed the apical expression and localization of fatty acid transporter FATP4 in the enteroids by immunofluorescence staining (Figure 5A). Next, fatty acid uptake was assessed using live-cell imaging.^43^ The fluorescent C12 analog, C1-BODIPY-C12, was rapidly taken up by AO enteroids and displayed a punctate intracellular distribution consistent with lipid droplet localization (Figure 5B). In contrast, BO enteroids showed predominantly passive intraluminal accumulation of C1-BODIPY-C12, with minimal intracellular punctuate staining in enterocytes (Figure 5C). This behavior suggests impaired intracellular distribution in the absence of proper FATP4 function. Co-staining with LipidTOX Deep Red confirmed colocalization of C1-BODIPY-C12 with neutral lipid droplets in AO enteroids (Figure 5D), consistent with FATP4-dependent metabolic trapping following acyl-CoA synthetase-mediated activation. Together, these findings demonstrate FATP4-mediated fatty acid uptake in live AO enteroids and support previous results in fixed enteroids.^19^ Both AO and BO enteroids also expressed other members of the FATP family (SLC27A1-3) at low *in vivo*-like levels (Figure S5; Table S6).

**Figure 5:**
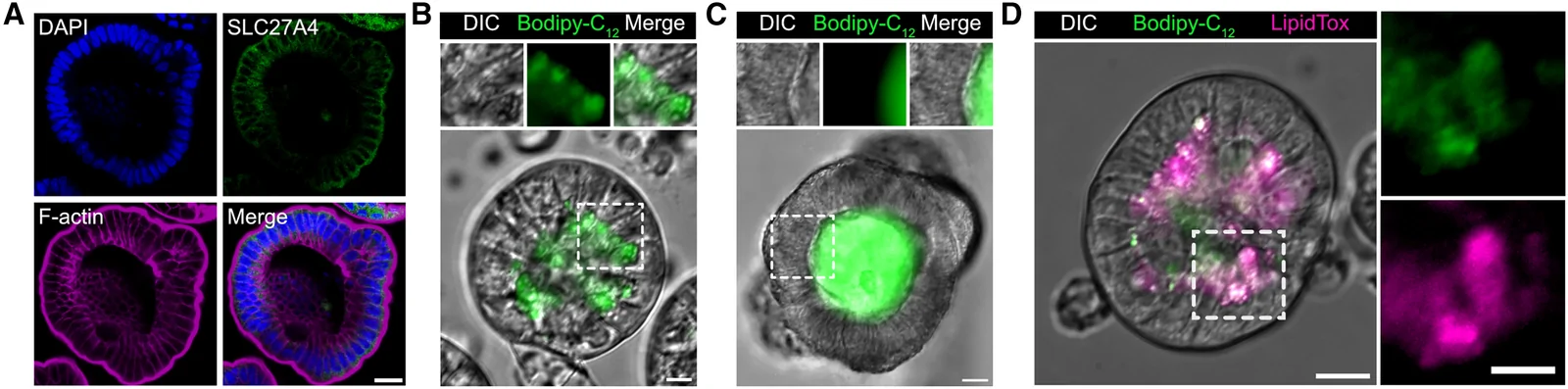
Fatty acid transport in 3D enteroids in suspension (A) Representative confocal images following staining with DAPI (nuclei; blue), SLC27A4 (solute carrier family 27 member 4, green) and phalloidin (F-actin; magenta), highlighting the expression pattern of the fatty acid transport protein in AO enteroids (donor Hu18-9jej). Scale bars, 20 μm. (B and C) AO and BO enteroids were incubated with fluorescent fatty acid analog C1-BODIPY-C12 for 10 min and imaged live using widefield fluorescence microscopy. The dashed square indicates the region shown at higher magnification. (B) C1-BODIPY-C12 accumulated in fluorescent lipid droplets in AO enteroids. Scale bars, 10 μm. (C) BO enteroids showed intraluminal accumulation of C1-BODIPY-C12. Scale bars, 20 μm. (D) AO enteroids (donor Hu18-9jej) were co-incubated with fluorescent fatty acid analog C1-BODIPY-C12 and LipidTOX-DR for 10 minto visualize lipid droplets. AO enteroids were imaged live using widefield fluorescence microscopy. The dashed square indicates the region shown at higher magnification. Scale bars, 20 μm; inset scale bars, 10 μm. (A–D) Enteroids from donor Hu18-9jej were used. See also Figure S5.

### The clinically important transporters Pgp and BCRP are functional in apical-out and basal-out enteroids

Of the nine transporters recommended for regulatory testing of drug candidates, Pgp/*ABCB1* and BCRP/*ABCG2* are most relevant in the small intestine.^42^ Pgp expression in enteroids was about 2-fold higher than in enterocytes and tissue (Figure 4C; Table S7), with correct apical localization confirmed by immunostaining (Figures 6A and 6B). To evaluate Pgp functionality, we developed a live-cell microscopy assay using Rho123, a fluorescent substrate for the apically located Pgp efflux transporter (Figures 6C–6I).^44^ In both AO and BO enteroids, Pgp efficiently effluxed the fluorescent substrate Rho123 into the medium, while the inhibitor elacridar (ELC) caused its intracellular retention (Figures 6C–6E). In BO enteroids, Rho123 accumulated in the lumen, consistent with luminal (apical) localization of Pgp. In the presence of ELC, efflux was reduced, leaving dye intracellularly in enterocytes (Figures 6H and 6I). Overall, BO enteroids showed an approximately 5-fold higher luminal fluorescence than AO, with some heterogeneity across the population as observed also in the live-cell integrity assay with LY (Figures 3D–3G).

**Figure 6:**
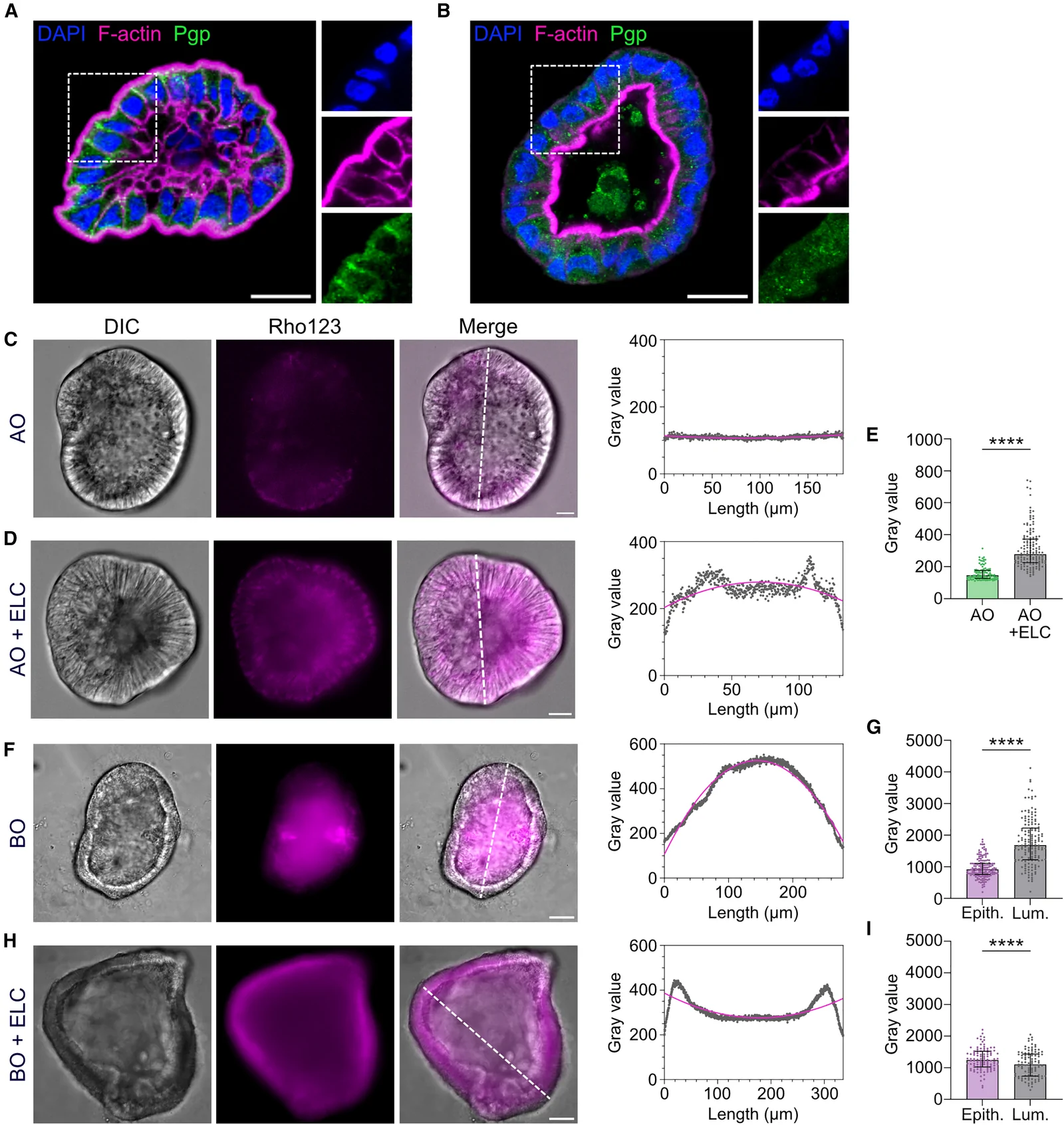
Real-time analysis of Pgp-mediated transport of Rho123 in 3D enteroids in suspension (A and B) Representative images for AO (A) and BO (B) enteroids illustrating nuclei (blue), Pgp (green), and F-actin (magenta). Scale bars, 20 μm. (C) Representative images from a population of *n* = 284 AO enteroids following incubation with Rho123 (10 μM) at 37°C for 1 h. The Rho123 fluorescence profile (right image) corresponds to the white line in the merged image. Scale bars, 20 μm. (D) In control experiments, AO enteroids were pre-treated with the Pgp inhibitor elacridar (ELC, 2 μM) for 1 h, then incubated with Rho123 (10 μM) and ELC (2 μM) for 1 h. Representative live-cell images are shown from a population of 227 enteroids, with the Rho123 fluorescence profile (right image) corresponding to the white line in the merged image. Scale bars, 20 μm. (E) Quantification of Rho123 fluorescence intensity between AO enteroids treated only with Rho123 (*n* = 284) and control AO enteroids treated with ELC and Rho123 (*n* = 227). Data are presented as median ± IQR, ∗∗∗∗*p* < 0.0001 (Mann-Whitney *U* test). Data are representative of one of two independent experiments. (F) Representative images of a population of *n* = 154 BO enteroids incubated with Rho123 (10 μM) at 37°C for 1 h. The fluorescence profile (right image) corresponds to the white line in the merged image. Scale bars, 20 μm. Data are representative of one of two independent experiments. (G) Quantification of Rho123 fluorescence intensity within BO enteroid epithelium and lumen (*n* = 154). Data are shown as median ± IQR, ∗∗∗∗*p* < 0.0001 (Wilcoxon signed-rank test). (H) BO enteroids pre-treated with ELC (2 μM) for 1 h, then incubated with Rho123 (10 μM) and ELC (2 μM) for 1 h. Representative images are shown from a population of *n* = 102 enteroids, and the fluorescence profile (right image) corresponds to the white line in the merged image. Scale bars, 20 μm. (I) Quantification of Rho123 fluorescence intensity in ELC-treated BO enteroid epithelium and lumen (*n* = 102). Data are shown as median ± IQR, ∗∗∗∗*p* < 0.0001 (Wilcoxon signed rank test). In (A–I) enteroids from donor Hu18-9jej were used.

Enteroid BCRP expression was comparable to that of freshly isolated enterocytes and tissue (Figure 4C; Table S7). BCRP was localized apically where it overlapped with the actin belt of the terminal web (Figure 7B). Functional analysis using a plate reader assay and the fluorescent BCRP substrate pheophorbide A (PhA)^45^ with or without the inhibitor KO143^46^ showed robust efflux. The AO enteroids had comparable efflux ratios to MDCK-hBCRP cells, which overexpress wild-type human BCRP (hBCRP, Figures 7A and 7C).^46^ This finding indicates that BCRP can effectively efflux its substrate from the AO enteroids.

**Figure 7:**
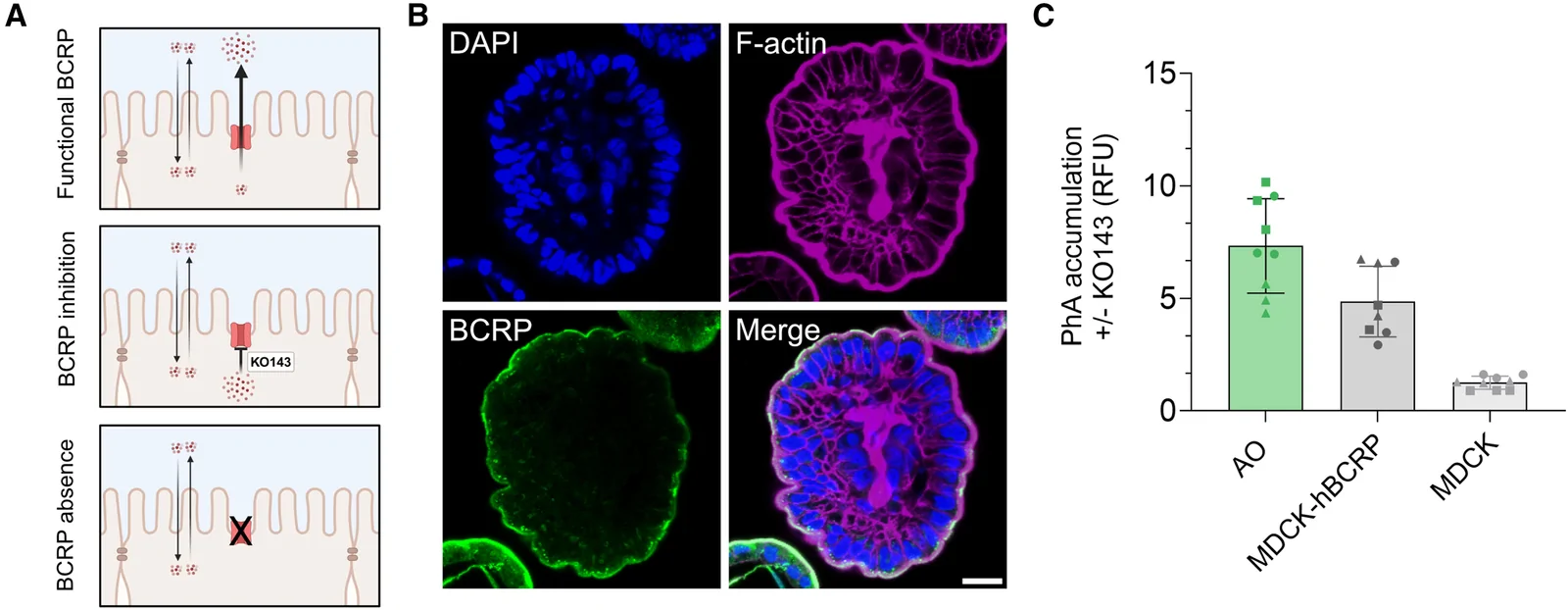
BCRP expression, localization and activity in AO enteroids in suspension (A) Schematic illustrating BCRP efflux under physiological function, inhibition or absence (e.g., knockout). (B) Representative confocal images following staining with DAPI (nuclei; blue), BCRP (green) and phalloidin (F-actin; magenta), highlighting the expression pattern of the BCRP transporter in AO enteroids (donor Hu18-9jej). Scale bars, 20 μm. (C) BCRP efflux in AO enteroids (donor Hu18-9jej) calculated by PhA accumulation; transporter substrate: PhA, transporter inhibitor: KO143. Results plotted as mean ± SD. Data are shown as triplicates in three independent experiments; data points of each independent experiment denoted as an individual shape.

### Apical-out and basal-out enteroids express key metabolizing enzymes at *in vivo* levels

Next, we investigated if the expression of central metabolizing enzyme families in the 3D enteroids reflects that observed in mature villus enterocytes. A total of 17 CYP isoenzymes of the CYP enzyme family, which mediate monooxidative reactions, were identified (Table S8). Among these, CYP3A4, the clinically most important CYP drug-metabolizing enzyme, which metabolizes more than 50% of all approved drugs,^47^ was expressed in AO and BO enteroids within 2-fold of those in primary enterocytes and tissues (Figure 4D; Table S8). As expected, other polymorphic CYP isoenzymes showed variable expression levels. For instance, CYP2C19 and CYP2C9 protein levels were up to 3-fold lower compared to primary enterocyte or jejunal tissue samples (Figure 4D; Table S8). By comparison, MBO had a lower expression of the essential CYPs (Figure 4D; Table S8).

In addition to CYP enzymes, other phase I enzymes, including carboxylesterases (CESs), which catalyze the hydrolysis of various substrates,^48^ showed comparable expression levels between enteroids and enterocytes (Table S9). CES1, known for hydrolyzing substrates with larger acyl groups, and the important and quantitatively dominating CES2 (CES2/CES1 ratios are 44 and 85 in AO enteroids and primary enterocytes, respectively), which targets smaller acyl groups, were both detected at *in vivo* levels in the AO and BO enteroid samples, but was several-fold lower in MBO (Table S9). In contrast, Caco-2 cells predominantly express CES1 rather than the intestinal isoform CES2.^49^ Important alcohol dehydrogenases involved in ethanol and formaldehyde metabolism (ADH4 and ADH5) and aldehyde dehydrogenases such as ALDH2 and ALDH1B1, which have critical roles in acetaldehyde metabolism and epithelial differentiation, respectively,^50^ were expressed in all three groups of enteroids (Table S9). Additionally, flavin-containing monooxygenases (FMOs) were found, revealing FMO5 as the most abundant isoform across all samples (Table S9). The high expression of FMO5 underscores its important role in metabolic processes within both the enteroids and native tissue.^51^^,^^52^

We next looked at phase II enzymes, which are essential for conjugation reactions that enhance metabolite solubility and elimination. Most phase II enzymes were quantified in the different samples. For instance, UDP-glucuronosyltransferases (UGTs), which catalyze the addition of glucuronic acid to compounds such as acetaminophen, morphine, and estrogens, showed comparable expression levels in AO and BO enteroids and enterocytes.^53^ Among 10 quantified UGTs, UGT1A1, UGT1A6, and the gut enriched UGT1A10 were expressed levels in suspended enteroids at levels comparable to or higher than *in vivo*, whereas UGT2B7 and UGT2B17 showed similar or lower expression (Figure 4E; Table S10). In contrast, the sulfotransferases (SULTs), which transfer sulfate groups to endogenous compounds and drugs, showed lower expression levels throughout (∼2- to 19-fold lower) compared to enterocytes and jejunal tissue (e.g., SULT1A1, SULT1A3, Figure 4F; Table S11).^54^ SULT2A1 showed a discrepancy between AO and BO, with BO having a 12-fold lower expression compared to mucosa, whereas AO was 1.5- to 2-fold lower than the *in vivo* reference. However, enzymes such as SULT1B1 were expressed at even lower levels (Figure 4F; Table S11). This pattern may reflect differential regulation of phase II enzymes *in vitro*, where glycolysis-supported glucuronidation is maintained, whereas sulfotransferase expression is reduced due to limited substrate exposure, cofactor availability, and absence of physiological regulatory signals. In general, the phase II enzymes were expressed at very low levels and sometimes not detected at all in the MBO enteroids.

Another important enzyme family, the glutathione S-transferase (GST) family, plays a central role in detoxification by conjugating glutathione to xenobiotics and reactive oxygen species.^55^ Of the GST enzymes, 12 were quantified, with GSTM1 and GSTT1 showing similar expression levels across all three enteroid culture conditions, enterocytes, and jejunal tissue (Table S12). The dominant GSTP1, which is often upregulated *in vitro* as part of elevated cellular stress, showed markedly higher expression in enteroids (∼2- to 5-fold higher) compared to *in vivo* levels. Finally, N-acetyltransferases (NAT1 and NAT2),^56^ which catalyze the acetylation of drugs and carcinogens, were expressed at levels comparable to those *in vivo* (Table S12). Together, these results further highlight the metabolic fidelity of AO and BO enteroids and their utility as models for studying intestinal metabolism. With the exception of GSTA1, the expression of GSTs and NATs in less differentiated MBO enteroids was on par with the other samples. This finding is consistent with GSTA1 upregulation during enterocyte differentiation and enrichment in mature epithelial cells.^57^

### Apical-out enteroids exhibit functional CYP3A4 metabolism and enable PBPK modeling

We next investigated the functional activity of CYP3A4, the most clinically relevant drug-metabolizing enzyme, using two orally administered probe drugs, terfenadine and midazolam, whose human pharmacokinetic profiles are strongly influenced by intestinal CYP3A4 metabolism (Figure 8; Figure S6, respectively). Furthermore, despite that both drugs are Pgp substrates, their absorption is not significantly affected by efflux due to their high intestinal permeability.^58^^,^^59^ Strong intracellular CYP3A4 staining was observed in the majority of enterocytes within the AO enteroids, with localization primarily along the cell borders (Figure 8A). Functionally, terfenadine, which is rapidly metabolized to fexofenadine by CYP3A4 and, in parallel, azacyclonol,^60^ was efficiently cleared from the medium when added to the enteroids. Fexofenadine simultaneously appeared in the medium (Figure 8B). The average intrinsic clearance (CL_int_) of AO enteroids was 2.6 μL × min^−1^ × (1×10^6^ cells)^−1^, which was about 8-fold lower than in freshly isolated jejunal enterocytes (Figure 8C). However, when normalized to CYP3A4 protein expression, the difference in CL_int_ was reduced to approximately 2-fold CL_int_ (Figure 8D).

**Figure 8:**
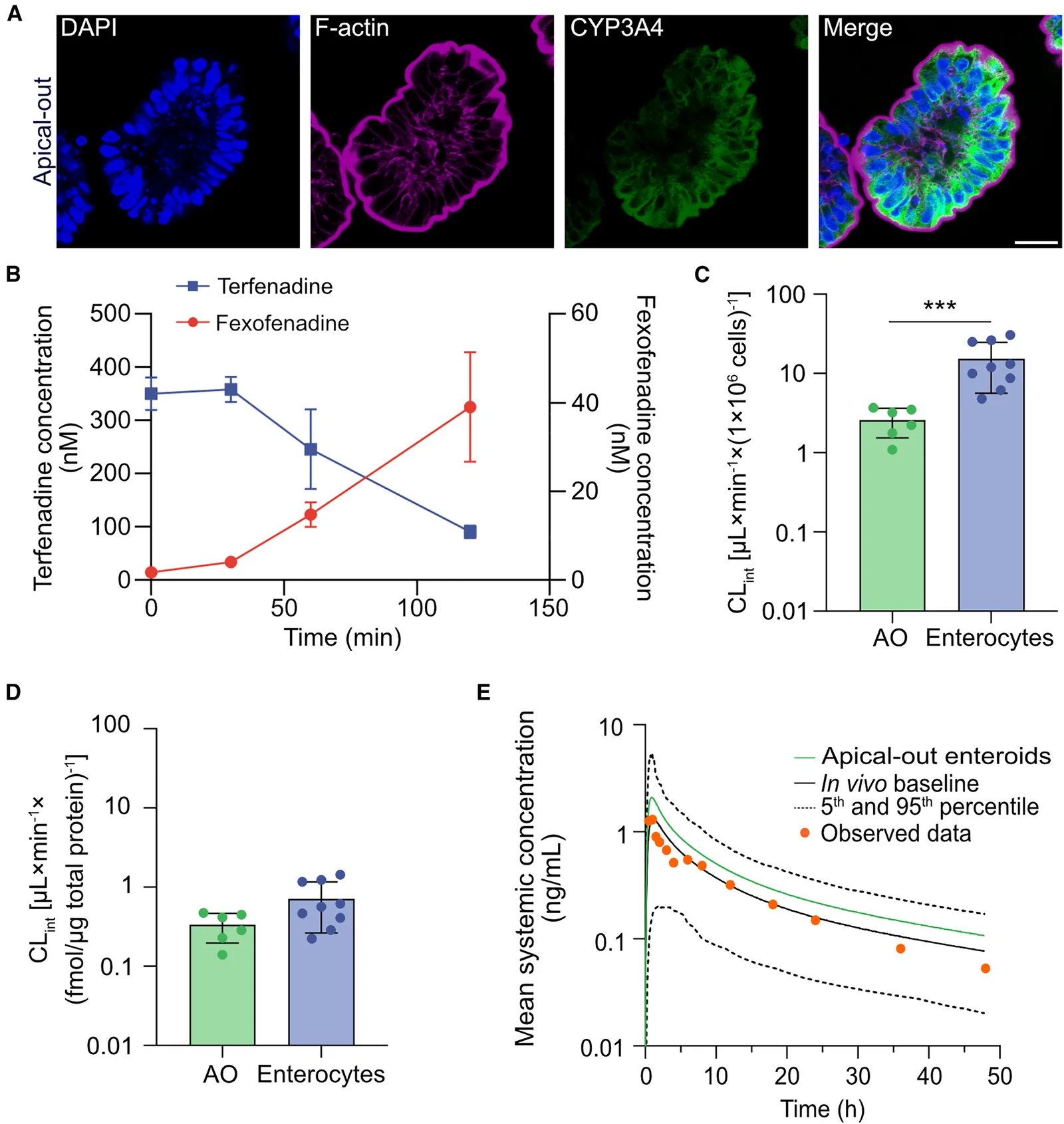
CYP3A4 metabolic activity in AO enteroids in suspension and PBPK simulation (A) Representative confocal images following staining with DAPI (nuclei; blue), CYP3A4 (green), and phalloidin (F-actin; magenta), highlighting the expression pattern of the CYP3A4 metabolic enzyme in AO enteroids (donor Hu18-9jej). Scale bars, 20 μm. (B) CYP3A4 metabolic activity in AO enteroids (donor Hu18-9jej) over a time period of 120 min. The concentration of the enzyme substrate terfenadine was monitored over time, as well as the formation of its metabolite fexofenadine. Data are shown as mean ± SD. (C) Intrinsic clearance of terfenadine in AO enteroids (donor: Hu18-9jej) and primary enterocytes (donors: Hu22-32jej, Hu23-1jej and Hu23-5jej). Data are shown as median ± IQR, ∗∗∗*p* < 0.001 (Mann-Whitney *U* test). (D) Corrected intrinsic clearance of terfenadine considering the differences in CYP3A4 protein expression in AO enteroids (donor Hu18-9jej) and primary enterocytes (donors: Hu22-32jej, Hu23-1jej and Hu23-5jej. Data are shown as median ± IQR. Mann-Whitney *U* test showed no statistically significant difference between groups. (E) Simulated mean pharmacokinetics profile of a 60 mg dose of terfenadine in 140 virtual males (20–50 years, black line) with its 5th and 95th percentile profile shown as dashed lines. The green line represents the PBPK model mean prediction for AO enteroids (log concentration). The orange dots depict mean observed data from Okerholm et al.^61^ See also Figure S6; Table S13.

To assess the translational relevance of this model, we performed PBPK simulations incorporating the enteroid-derived metabolism parameters. The baseline PBPK model of terfenadine contained observed *in vivo* PK data for a 60 mg single oral dose in a virtual population of 140 adult males (ages 20–50, Figure 8E). The simulation parametrized with enteroid data showed that the predicted plasma concentrations closely matched clinical data and remained within the 95% confidence interval of the baseline simulation (Figure 8E). The model predicted a fraction escaping intestinal metabolism (F_g_) of 0.46, compared to the baseline value of 0.32 for the population. Similar results were obtained when the enteroid-derived metabolism was used to predict the pharmacokinetics of midazolam (Figure S6). These results demonstrate that AO enteroids possess functional CYP3A4 activity that supports quantitative predictions of the impact of intestinal metabolism on systemic drug exposure. The ability to recapitulate the most important metabolic processes and support PBPK modeling, highlights the utility of AO enteroids as a physiologically relevant *in vitro* platform for evaluating intestinal drug metabolism.

## Discussion

Absorption of ingested materials in the human intestine is mediated primarily by differentiated epithelial cells at the villus tips, where absorptive enterocytes constitute approximately 95% of the intestinal epithelial cell population.^62^ The goal of this study was to establish suspended organoid cultures enriched in such differentiated absorptive cells and equipped with both physical and metabolic barrier functions. These enteroids were designed to mimic the selective epithelial properties that enable efficient absorption of essential nutrients via solute transporters while limiting exposure to toxicants through efflux systems, such as ABC transporters, and xenobiotic-metabolizing enzymes.

In contrast to many existing enteroid protocols that aim to recapitulate the full spectrum of epithelial cell types along the crypt-villus axis, our approach was specifically tailored to model villus-tip enterocytes.^63^^,^^64^ As a consequence, the resulting enteroids exhibited a predominance of morphologically and biochemically differentiated absorptive cells, with a low abundance of other epithelial cell types. A limitation of this cellular composition is that it precludes the formation of a continuous extracellular mucus barrier composed of secreted gel-forming mucins, which are typically produced by goblet cells. Although recent protocols have been developed to increase the proportion of secretory cell types, including goblet cells, to approximately 5% in human enteroids, such approaches often require enhanced stemness as a preconditioning step to promote cellular diversity.^64^ This shift, however, reduces the frequency of fully differentiated absorptive cells with dense microvilli and an *in vivo*-like glycocalyx barrier, features that are integral to the functional villus-tip epithelium (Table S3).

Enhanced differentiation toward the absorptive enterocyte lineage was achieved by extending the culture period in differentiation medium to up to six days. This approach resulted in reduced expression of stem cell and proliferation markers and a concomitant increase in markers characteristic of mature absorptive cells, including the brush border enzymes ANPEP and SI, to levels approaching those observed in human jejunal tissue (Figure 1). Given the short lifespan of enterocytes *in vivo*, prolonged culture raises concerns regarding terminal differentiation and excessive apoptosis. However, the expression of late apoptotic markers remained comparable to that observed in human jejunal tissue, indicating that apoptosis was maintained within physiologically acceptable limits. Consistent with previous reports, differentiated BO enteroids exhibited characteristic crypt-like structures that protruded into the surrounding medium and increased in size over time.^17^ This phenotype agrees with the residual, albeit low, expression of stem cell and proliferation markers detected in these cultures (Figures 1E and S1). In contrast, the small, enclosed luminal space and largely spherical morphology of differentiated AO enteroids impose physical constraints that limit the formation of inward-folding crypt-like domains, thereby contributing to lower proliferative activity. In addition, the outward-facing apical membrane of AO enteroids is exposed to increased shear stress, a known driver of enterocyte differentiation and maturation via mechanotransduction, further reinforcing a villus-like phenotype.^65^

Despite these architectural differences, differentiated AO and BO enteroids displayed comparable expression of differentiation markers, microvilli-associated proteins, and tight-junction components, consistent with the acquisition of a shared mature epithelial phenotype.^65^ This conclusion was supported by immunofluorescence, TEM, and SEM analyses, together with proteomic data, which collectively demonstrated a well-developed membrane barrier characterized by densely packed, crosslinked microvilli, appropriately localized and functional efflux proteins, and a distinct, well-organized glycocalyx (Figure 2).

Similarly, TEM analyses combined with protein expression data revealed a well-developed paracellular barrier, including a fully functional junctional complex with clearly distinguishable tight and adherens junctions (Figure 2A). Together, these results indicate that the enteroids achieved a level of differentiation sufficient to enable functional assessment of epithelial barrier integrity, transport processes, and metabolic activity. The *in vivo*-like expression of tight and adherens junction proteins suggested the presence of a fully functional epithelial tight-junction barrier. Consistent with this interpretation, apoptotic cells observed by SEM and TEM retained intact tight-junction connections, indicating preservation of barrier integrity even in cells undergoing apoptosis.^66^

Previous investigations of enteroid barrier function have commonly relied on 4-kDa FITC-dextran (FD4) as a permeability probe.^67^^,^^68^ However, due to its relatively large molecular size, FD4 lacks the sensitivity required to detect subtle defects in barrier integrity that permit leakage of small solutes while excluding larger macromolecules. Accordingly, clinical studies of intestinal permeability frequently employ smaller hydrophilic probes, such as lactulose (342 Da) and mannitol (182 Da), which more accurately reflect paracellular permeability.^69^ In addition, the molecular weight of most orally administered drugs falls within the range of approximately 200–500 Da.^70^

Based on these considerations, we selected LY as a fluorescent small-molecule probe and quantified its exclusion from AO and BO enteroids using live-cell microscopy. Both AO and BO enteroids retained functional tight-junction barrier properties throughout the duration of the study. However, permeability to LY appeared more heterogeneous than suggested by protein expression analyses and imaging of fixed organoids. This variability is consistent with previous reports describing heterogeneity in AO enteroid permeability.^36^^,^^71^ Nevertheless, the entire AO enteroid population could be analyzed directly in the LY exclusion assay, as all AO enteroids consistently excluded LY throughout the duration of the experiment. In contrast, a small subpopulation of immature, stem-like BO enteroids lacked appropriate barrier properties and was therefore excluded from analysis. The remaining major population of differentiated BO enteroids demonstrated consistent entrapment of the marker in their luminal space (Figure 3). We therefore tentatively conclude that both AO enteroids and differentiated BO enteroids following the omission of budded, stem-like immature structures in the BO configuration—exhibit barrier properties that closely approximate those observed *in vivo*, supporting their suitability for direct studies of intestinal epithelial barrier function.

The high expression of multiple nutrient and drug transporters in the suspended enteroids prompted us to assess the functional activity of crucial transport pathways. As an initial readout of lipid absorption, we used the fluorescent fatty-acid probe C1-BODIPY-C12, which serves as a proxy for long-chain fatty-acid uptake and is predominantly transported in the human intestine by the highly expressed fatty-acid transport protein FATP4.^19^ In AO enteroids, targeted accumulation of C1-BODIPY-C12 in intracellular neutral lipid droplets was observed exclusively in enteroids exhibiting appropriate apical localization of FATP4. This pattern is most consistent with directional fatty-acid uptake coupled to long-chain acyl-CoA synthetase activity associated with FATP4-mediated transport (Figures 5B and 5C).^72^ In contrast, BO enteroids displayed only passive accumulation of the probe, with fluorescence largely confined to the luminal compartment. Together, these findings are consistent with polarized, apical-to-intracellular fatty-acid transport mediated by FATP4 and highlight the functional relevance of epithelial orientation for lipid uptake in this system.^73^

Understanding the mechanisms governing intestinal drug transport is critical for oral drug development. To this end, *in vitro* models of the intestinal epithelium, most notably Caco-2 monolayers, are routinely employed despite well-recognized limitations.^8^ Of particular clinical relevance are the ABC transporters Pgp and BCRP, which mediate the efflux of drugs and xenobiotics back into the intestinal lumen.^42^^,^^74^ We first assessed Pgp function in differentiated enteroids using live-cell microscopy and the established Pgp substrate Rho123.^44^ As observed in the LY barrier integrity assays, efflux activity exhibited variability across the enteroid population; nevertheless, the spatial resolution of the assay enabled robust visualization of transporter activity at the level of individual enteroids. However, imaging-based analysis of single enteroids is labor-intensive and will require further automation for routine application. To evaluate BCRP function, we therefore employed a higher-throughput plate-reader assay in AO enteroids using PhA, a well-established BCRP-specific fluorescent substrate.^46^ Using this approach, functional BCRP activity was readily detected. As a comparison, we used an engineered MDCK model with CRISPR-Cas9 knockout of endogenous canine MDR1 (cMDR1)^75^ and replacement with hBCRP,^46^ serving as a reference for qualitative assessment of BCRP activity. Together, these data demonstrate that both clinically important intestinal ABC transporters, Pgp and BCRP, are functionally expressed in jejunal enteroids. While drug-drug interaction studies involving these transporters are regulatory requirements, their impact on intestinal drug absorption is generally modest, with most reported clinical effects being small (typically <2-fold), as exemplified by digoxin as a sensitive probe substrate.^76^

Similarly, first-pass metabolism is usually dominated by the liver, but intestinal metabolism can nevertheless make a major contribution for selected orally administered drugs, especially CYP3A substrates, and may, in some cases, approach or even exceed hepatic first-pass extraction, emphasizing the relevance of studying metabolism in enteroids.^77^ Thus, the bioavailability and the clinical efficacy of orally administered drugs can be influenced by first-pass metabolism in both the intestinal epithelium (F_g_) and liver (F_h_). While F_h_ can be predicted from *in vitro* culture of primary human hepatocytes, predictive cell culture models of first-pass metabolism in the gut (F_g_) have not been available. Although cultures of primary human hepatocytes have become a gold standard for prediction of first-pass metabolism in the liver (F_h_) after decades of optimization, the development of the corresponding primary intestinal epithelial cell cultures is lagging, hampered by their short half-life in culture.^4^^,^^5^ Subcellular fractions such as liver microsomes have an even longer history in predictions of hepatic drug metabolism, while intestinal microsomes are equally useful but not generally available.^78^

Recent studies using intestinal enteroids, particularly after seeding into 2D monolayers or alternative formats,^24^^,^^26^ have demonstrated promise for modeling intestinal drug metabolism, although with variable success in recapitulating *in vivo* metabolic activity.^24^^,^^26^^,^^79^ In some systems, induction via the vitamin D receptor has been employed to enhance CYP3A4 expression and metabolism, whereas in other studies, *in vivo*-like CYP3A4-mediated metabolism was achieved without such induction, and F_g_ was estimated.^24^^,^^26^^,^^79^ In the present study, we demonstrate that suspended 3D jejunal enteroids in the AO configuration can accurately capture intestinal metabolism and its impact on systemic drug exposure. Using CYP3A4-mediated metabolic clearance of terfenadine and midazolam, we predicted human pharmacokinetics that were consistent with clinical observations and well within the confidence intervals of a simulated human virtual population.^61^ These findings position human jejunal AO enteroids as a physiologically relevant and predictive platform for investigating intestinal metabolism and presystemic drug clearance. Future studies extending this approach to a broader range of substrates and toxicants will further define the predictive performance and translational utility of this model.

Transporters and metabolic enzymes frequently share substrates or operate in coordinated, sequential pathways.^80^ For example, a drug or toxicant may be taken up by an SLC transporter, metabolized by one or more enzymes within the enterocyte, and subsequently effluxed as a parent compound or metabolite by an ABC transporter.^81^ A well-documented example of such functional interplay is the coordinated action of Pgp and CYP3A4,^82^ which are co-expressed and function at near *in vivo* levels in the differentiated enteroids described here. This creates opportunities to investigate transporter-enzyme interactions that have historically been studied primarily in artificial heterologous expression systems.

Furthermore, the ability to maintain 3D cultures for extended periods enables detection of slowly formed metabolites that may be missed in short-term assays using primary enterocytes or intestinal microsomes. As demonstrated for Pgp in BO enteroids, these metabolites that serve as substrates for apically located ABC transporters can accumulate within the enclosed luminal space, facilitating their detection, identification, and characterization. Finally, many of the quantified metabolic enzymes have detoxifying functions. Their expression at physiologically relevant levels supports the use of the 3D suspended enteroids for predictive toxicity studies and so extends their application beyond pharmacokinetics into drug safety evaluation.

In conclusion, we provide, for the first time, a comprehensive quantitative proteomic characterization of human jejunal 3D enteroids in distinct configurations. We demonstrate that differentiated jejunal enteroids in suspension, which represent the principal absorptive region of the human intestine, closely recapitulate the proteomic landscape of mature absorptive epithelial cells located at the villus tips of the human jejunum. These enteroids form an intact epithelial barrier and exhibit essential functional properties, including nutrient uptake, polarized drug transport, and CYP3A4-mediated metabolism, thereby constituting a versatile and reproducible “mini-gut” system.

Collectively, these findings establish the translational relevance of both AO and BO jejunal enteroids in suspension as physiologically meaningful human models for investigating intestinal epithelial function. Their *in vivo*-like structural and functional characteristics support broad applicability not only in mechanistic studies of normal and diseased intestine, but also in investigations of the transport and metabolism of food constituents, drugs, and other xenobiotics in a physiologically relevant context.

### Limitations of the study

One limitation of this study is that the enteroid model was designed to recapitulate absorptive villus enterocytes rather than the full cellular complexity of the jejunal epithelium; accordingly, secretory cell types such as goblet cells are underrepresented. In addition, the cultures are not fully homogeneous, which may affect interpretation of population-based functional assays. The system is also experimentally demanding and has not yet been adapted to automated small-scale formats, which currently limits throughput. Residual Matrigel, particularly in BO enteroids, may further interfere with some imaging-based assays due to nonspecific binding of antibodies to extracellular matrix proteins. Finally, although the optimized differentiation conditions yielded strong resemblance to native jejunal epithelium, the culture conditions are unlikely to capture all *in vivo* maturation cues, leaving room for further refinement.

## Resource availability

### Lead contact

Requests for further information and resources should be directed to and will be fulfilled by the lead contact, Per Artursson (per.artursson@uu.se).

### Materials availability

This study did not generate new unique reagents.

### Data and code availability

• The mass spectrometry proteomics data have been deposited at ProteomeXchange Consortium via the PRIDE partner repository as dataset identifier PRIDE: PXD067571 and are publicly available as of the date of publication.
• This paper does not report original code.
• Any additional information required to reanalyze the data reported in this paper is available from the lead contact upon request.

## Acknowledgments

This research was funded by the 10.13039/501100004359Swedish Research Council grant no. 2017-02243 (DLW, PMH), 2017–01951 (PA), 2020–05186 (PA), Swedish Foundation for Strategic Research grant no. FFL18-0165 (MES), 10.13039/501100001858VINNOVA (2019–00048, DH, and 2024–03851, RH) for the Swedish Drug Delivery Center (SweDeliver), the YLSY doctoral scholarship program from the Republic of Turkey, Ministry of National Education (MC), the European Union's Horizon 2020 research and innovation program under the Marie Skłodowska-Curie grant agreement no. 956851 (COLOTAN, PA and RH), the European Union's Horizon 2020 research and innovation program under grant agreement No 964537 for RISK-HUNT3R: RISK assessment of chemicals integrating Human-centric Next Generation Testing strategies promoting the 3Rs (PA and PL), and the European Union’s Horizon 2021 research and innovation action (RIA) for GENEGUT (GA 101057491, PA and MH). Views and opinions expressed are however those of the author(s) only and do not necessarily reflect those of the European Union or the European Health and Digital Executive Agency (HaDEA). Neither the European Union nor the granting authority can be held responsible for them. The authors are grateful for financial support from Selander’s Foundation (DLW, PMH), OE and Edla Johansson’s Science Foundation (DLW, PMH) and ALF (ALF-899261, DLW, PMH). The authors acknowledge the facilities and technical assistance of the Umeå Center for Electron Microscopy (UCEM) at the Chemical Biological Center (KBC), Umeå University, a part of the National Microscopy Infrastructure NMI (VR-RFI 2019–00217). We thank Dr. Evgeniya Mickols (Uppsala University) for her contribution to the design of the graphical abstract included in this manuscript.

## Author contributions

Conceptualization: M.E.S., M.H., and P.A.; methodology: F.T., M.C., M.L.D.M., D.S.V., A.C.C.L., R.H., I.G., P.L., J.E., D.H., M.E.S., M.H., and P.A.; investigation: F.T., M.C., M.L.D.M., D.S.V., A.C.C.L., R.H., I.G., P.L., J.E., and D.H.; formal analysis: F.T., M.C., M.L.D.M., D.S.V., A.C.C.L., R.H., I.G., P.L., J.E., and D.H.; resources: M.Su., M.sk., P.M.H., D.L.W., M.E.S., and P.A.; visualization: F.T., M.C., and M.H.; supervision: M.K., P.L., M.E.S., M.H., and P.A.; project administration: M.Su., M.sk., P.M.H., D.L.W., M.E.S., M.H., and P.A.; funding acquisition: P.M.H., D.L.W., M.E.S., M.H., and P.A.; writing – original draft: F.T., M.C., D.S.V., M.H., and P.A.; writing – review and editing: all authors.

## Declaration of interests

The authors declare no competing interests.

## Declaration of generative AI and AI-assisted technologies in the writing process

During the preparation of this work, the authors used Microsoft Copilot GPT -5 as a language tool for the literary refinement of the text. The authors have since reviewed and edited the content as needed and take full responsibility for the content of the publication.

## STAR★Methods

### Key resources table

**Table:** 

REAGENT or RESOURCE	SOURCE	IDENTIFIER
**Antibodies**		

Mouse anti-zonula occludens	Thermofisher Scientific	Cat #: 33–9100 RRID: AB_2533147
BCRP/ABCG2 antibody [EPR21122]	Abcam	Cat #: ab229193 RRID:AB_3752998
CYP3A4 Monoclonal Antibody (3H8)	Thermofisher Scientific	Cat #: MA5-17064 RRID: AB_2538535
P-Glycoprotein Monoclonal Antibody (C219)	Thermofisher Scientific	Cat #: MA1-26528 RRID: AB_795165
Anti-SLC27A4/FATP4 antibody [EPR17319] - C-terminal	Thermofisher Scientific	Cat #: ab199719 RRID: AB_2716563
Goat anti-Rabbit IgG (H + L) Cross-Adsorbed Secondary Antibody, Alexa Fluor™ 488 - A-11008	Thermofisher Scientific	Cat #: A-11008 RRID: AB_143165
Goat Anti-Mouse IgG H&L (Alexa Fluor® 488)	Abcam	Cat #: ab150113 RRID: AB_2576208
Donkey anti-Rabbit IgG (H + L) Highly Cross-Adsorbed Secondary Antibody, Alexa Fluor™ 568 - A10042	Thermofisher Scientific	Cat #: A10042 RRID: AB_2534017

**Biological samples**		

Jejunal tissue Hu22-2jej	This study	N/A
Jejunal tissue Hu22-3jej	This study	N/A
Jejunal tissue Hu22-5jej	This study	N/A
Jejunal tissue Hu21-7jej	This study	N/A
Jejunal tissue Hu21-14jej	This study	N/A
Enterocytes Hu21-7jej	This study	N/A
Enterocytes Hu21-14jej	This study	N/A
Enterocytes Hu22-3jej	This study	N/A
Enterocytes Hu22-5jej	This study	N/A
Enterocytes Hu22-32jej	This study	N/A
Enterocytes Hu23-1jej	This study	N/A
Enterocytes Hu23-5jej	This study	N/A

**Chemicals, peptides, and recombinant proteins**		

IntestiCult™ Organoid Growth Medium (Human) “OGM”	STEMCELL TECHNOLOGIES	Cat #: 06010
IntestiCult™ Organoid Differentiation Medium (Human) “ODM”	STEMCELL TECHNOLOGIES	Cat #: 100–0214
Corning® Matrigel® Growth Factor Reduced (GFR) Basement Membrane Matrix, LDEV-free	Corning	Cat #: 354230
Gentle Cell Dissociation Reagent	STEMCELL TECHNOLOGIES	Cat #: 100–0485
Cell Recovery Solution	Corning	Cat #: 354253
Penicillin-Streptomycin (Pen Strep)	Gibco	Cat #: 15070063
DMEM F12	Gibco	Car #: 21041025
DMEM GlutaMAX™	Gibco	Cat #: 21885–025
Fetal Bovine Serum (FBS)	Gibco	Cat #: A5256701
Geneticin	Gibco	Cat #: 10131027
ROCK-inhibitor (Y-27632)	STEMCELL TECHNOLOGIES	Cat #: 72304
HBSS (10×), calcium, magnesium, no phenol red	Gibco	Cat #: 14065056
Alexa Fluor™ 660 phalloidin	Thermofisher Scientific	Cat #: A22285
Dimethyl sulfoxide (DMSO)	Sigma Aldrich	Cat #: D8418
Rhodamine 123	Sigma Aldrich	Cat #: R8004
Lucifer Yellow dilithium salt	Biotium	Cat #: 80015
Pheophorbide A	Sigma Aldrich	Cat #: SML1218
C1-BODIPY-C12 500/510	Thermofisher Scientific	Cat #: D3823
HCS LipidTOX™ Deep Red Neutral Lipid Stain	Thermofisher Scientific	Cat #: H34477
Elacridar	Sigma Aldrich	Cat #: SML0486
KO143 hydrate	Merck	Cat #: K2144
Sodium butyrate	Merck	Cat #: 8.17500
VECTASHIELD® Vibrance™ Antifade Mounting Medium with DAPI	Vector Laboratories	Cat #: H-1800–10
Ethylenediaminetetraacetic acid (EDTA)	MP Biomedicals	Cat #: 2820549
Bovine Serum Albumin (BSA)	Sigma Aldrich	Cat #: A9418
BSA Fraction V (7.5%)	Gibco	Cat #: 15260037
Glutaraldehyde	TAAB Laboratories	Cat #: G002
PIPES	Sigma Aldrich	Cat #: P6757
Egtazic acid (EGTA)	Merck	Cat #: E3889
4-(2-hydroxyethyl)-1-piperazineethanesulfonic acid (HEPES)	Sigma Aldrich	Cat #: H3375
MgCl_2_ × 6H_2_O	Sigma Aldrich	Cat #: M9272
Sodium cacodylate	Sigma Aldrich	Cat #: C0250
Potassium ferricyanide	Sigma Aldrich	Cat #: 702587
Tannic acid	EMS	Cat #: 21700
Uranyl acetate	TAAB Laboratories	Cat #: U001/S/1
Spurr’s resin	TAAB Laboratories	Cat #: S024/D
4% Paraformaldehyde (PFA)	Thermofisher Scientific	Cat #: J19943.K2
Dibasic sodium phosphate	Thermofisher Scientific	Cat #: 424375000
Monobasic sodium phosphate	Sigma Aldrich	Cat #: S0751
Sodium bicarbonate	Sigma Aldrich	Cat #: S5761
Saponin	Thermofisher Scientific	Cat #: S038048
Donkey serum	Merck	Cat #: D9663
Phosphate buffered Saline (PBS) w/o Ca^2+^/Mg^2+^	Gibco	Cat #:14190–094
Krebs-Henseleit buffer (Ca^2+^/Mg^2+^ −/−)	Sigma Aldrich	Cat #: K3753
Dithiothreitol (DTT)	Sigma Aldrich	Cat #: R0861
Percoll®	Sigma Aldrich	Cat #: GE17-0891-02

**Critical commercial assays**		

Acridine orange and propidium iodide (AOPI) viability assay	Nexcelom	Cat #: CS20106-5 ML
Reagent A100 lysis buffer	Chemometec	CAT #: 910–0003
Reagent B stabilizing buffer	Chemometec	Cat #: 910–0002

**Deposited data**		

Mass spectrometry global proteomics	Deposited to ProteomeXchange via PRIDE^83^ebi.ac.uk/pride/	PRIDE: PXD067571

**Experimental models: Organisms/strains**		

Hu18-9jej human jejunal enteroids	Geiser et al.^84^	N/A
Hu22-2jej human jejunal enteroids	van Rijn and Lopes et al.^25^	N/A
Hu22-3jej human jejunal enteroids	This study	N/A
Hu22-5jej human jejunal enteroids	This study	N/A

**Experimental models: cell lines**		

MDCK^cMDR1 KO^	Simoff et al.^75^	RRID: CVCL_A2JX
MDCK-hBCRP^cMDR1 KO^	Wegler et al.^46^	RRID: CVCL_A2KC

**Software and algorithms**		

Graphpad Prism 10	graphpad.com	RRID:SCR_002798
Fiji ImageJ v. 1.54j	Schindelin et al.^85^	ImageJ.net/software/fiji/ RRID:SCR_002285
R v. 4.3.3	r-project.org/	RRID: SCR_001905
RStudio v. 2025.09.2 + 418	posit.co/	RRID:SCR_000432
DEQMS R package v. 1.28.0	Zhu et al.^86^	https://doi.org/10.18129/B9.bioc.DEqMS bioconductor.org/packages/DEqMS/ RRID:SCR_025605
SimCYP v. 23	https://www.simcyp.com/	RRID:SCR_003944
MaxQuant v. 2.7.5.0	Tyanova et al.^87^	RRID:SCR_014485 maxquant.org/
clusterProfiler 4.10.1	Wu et al.^88^	https://doi.org/10.18129/B9.bioc.clusterProfiler bioconductor.org/packages/release/bioc/html/clusterProfiler.html RRID:SCR_016884
Velox v. 2.14.1.40	Thermofisher Scientific	Cat #: VELOX

**Other**		

24-well Clear TC-treated Multiple Well Plates	Corning	Cat #: 3524
24-well Clear Flat Bottom Ultra-Low Attachment Multiple Well Plates	Corning	Cat #: 3473
4tude transparent bottom plate	Azenta life sciences	Cat #: 4ti-0243
Corning costar transparent bottom plate 96w	Corning	Cat #: 3603
25 μm strainers	Funakoshi	Cat #: HT-AMS-12502
40 μm strainers	Funakoshi	Cat #: HT-AMS-14002

### Experimental model and study participant details

#### Human participants and primary tissue

Samples from human jejunum were collected from subjects with obesity undergoing Roux-en-Y gastric bypass surgery at the Uppsala University Hospital, Uppsala, Sweden. Subjects followed a low-calorie diet for three weeks prior to surgery and did not suffer from inflammatory or infectious bowel diseases at the time of surgery. Subjects diagnosed with type I or II diabetes were excluded from the study. Samples were taken 60 cm distal to the ligament of Treitz. Group level demographic information on the donor tissue samples is summarized in Table S1. All donors gave written informed consent. Samples were pseudonymized before proceeding with further analysis to protect patient identities and personal details. The study was reviewed and approved by local governing body (Etikprövningsmyndigheten, Sweden). Mucosal tissue preparations, jejunal enteroid establishments, and experimental work on those cultures was conducted under ethical permit number 2010–157 with addenda 2010–157-1 and 2020–05754. From April 2023, additional experimental enteroid work was also covered by ethical permit number 2023–01524-01. Jejunal tissues were received from nine different donors. No formal randomization was used to allocate donor-derived samples to experimental groups. The tissue samples were either snap frozen in liquid nitrogen and stored at −150°C, pending global proteomics analysis, or used immediately for isolation of primary jejunal enterocytes or adult jejunal stem cell-containing crypts for enteroid establishment. Sex was not evaluated as a biological variable in this study and therefore, potential sex-associated differences cannot be excluded and represent a limitation in terms of study generalizability.

#### Primary cell cultures: Human enterocytes and intestinal enteroids

Primary jejunal enterocytes were isolated directly from human tissue (see method details) cells were enriched via density gradient centrifugation and maintained in appropriate culture medium prior to experimental use. Enterocytes were derived from multiple donors (*n* = 7). Epithelial stem cell-containing crypts were isolated from human tissue and used to establish patient-derived 3D enteroids, as described in the method details section. Enteroids were cultured in Matrigel with defined growth media and maintained under standard conditions (37°C, 5% CO_2_), with regular passaging and differentiation prior to experimental use. Apical-out (AO) enteroids were generated by polarity reversal in suspension culture to enable direct access to the apical epithelial surface. Enteroids from multiple donors (*n* = 4) and passages 4–20 were used across experiments.

#### Cell lines

Madin-Darby canine kidney (MDCK) cell lines MDCK^cMDR1 KO^ ^75^ and MDCK-hBCRP^cMDR1 KO^ ^46^ were used as reference models for transport and efflux studies. Cells were maintained in DMEM supplemented with fetal bovine serum and antibiotics, with geneticin selection applied to MDCK-hBCRP^cMDR1 KO^ cells.^46^ The cell lines were originally obtained from the American Type Culture Collection (ATCC) and were authenticated by the supplier prior to distribution. No additional cell line authentication was performed in our laboratory. All cell cultures were routinely screened for mycoplasma contamination, and the MDCK cell lines used in this study tested negative for mycoplasma contamination.

### Method details

#### Primary enterocyte isolation from jejunal tissue

Intestinal samples (2–4 cm long pieces of jejunum) were collected in 50 mL tubes with oxygenated, ice-cold, calcium- and magnesium-free Krebs-Henseleit buffer (Sigma-Aldrich, St. Louis, MO, USA). Samples were immediately transported to the laboratory. The jejunal samples were opened along the mesentery and the mucosa was excised along the mucosa muscularis. The isolated mucosa was carefully washed three times in cold Krebs-Henseleit buffer and cut into small pieces (approximately 0.5–1 cm^2^). Primary enterocytes were dissociated by incubating 0.5–1 cm^2^ pieces of tissue in Krebs-Henseleit buffer supplemented with dithiothreitol (DTT, 1 mM, Sigma-Aldrich) and ethylenediaminetetraacetic acid (EDTA, 3 mM, Sigma Aldrich). Briefly, tissue samples amounting to approximately 1 g were first shaken on ice for 30 min on a microplate shaker at rpm 200–300 (VWR, Stockholm, Sweden), and then shaken at 37°C for 60 min. Mucus, larger tissue pieces, cell aggregates, and large debris were removed from the supernatant by filtering through a cell strainer (70 μm pore size, mini cell strainer, Funakoshi, Tokyo, Japan). To enrich for epithelial cells and remove blood cells, cell suspensions were centrifuged in Percoll (Sigma-Aldrich) density gradients [20%, 40% Percoll in Krebs-Henseleit buffer with 2% bovine serum albumin (BSA, Sigma-Aldrich)] at 10,000 *g* for 10 min, followed by washing with Krebs-Henseleit buffer with 2% BSA. The enriched cells were washed twice with 10 mL Krebs-Henseleit and resuspended in ice-cold Dulbecco′s Modified Eagle′s Medium (DMEM, Gibco, Thermo Fisher Scientific, Waltham, MA, USA). Viability was determined to be >85% by acridine orange and propidium iodide (AOPI) method in a Nexcelom Cellometer Vision (Nexcelom Bioscience, Lawrence, MA, USA). Jejunal tissues were from five different donors (Hu22-2jej, Hu22-3jej, Hu22-5jej, Hu21-7jej, and Hu21-14jej); enterocytes were from four of these (Hu22-3jej, Hu22-5jej, Hu21-7jej, and Hu21-14jej).

#### Adult jejunal epithelial crypt isolation

Stem cell-derived enteroid lines were established as described previously.^43^^,^^84^ Briefly, freshly isolated jejunal tissue was transferred to ice-cold phosphate buffered saline (PBS, Thermo Fisher Scientific) and placed on a Styrofoam cushion. PBS was used to remove particles, and the muscle layer was removed with scissors. An approximately 6 mm tissue piece was taken from the mucosa, rinsed with PBS, minced using surgical scissors, and passed through a 1 mL pipette tip. After centrifugation at 300 × *g* for 5 min and washing with cold PBS, the minced tissue was incubated in Gentle Cell Dissociation Reagent (STEMCELL technologies, Vancouver, BC, Canada) under gentle shaking on ice using a 3D nutating mixer (20 rpm, VWR) for 30 min. After a second centrifugation (300 × *g*, 5 min) and resuspension in cold DMEM F12 (Gibco, Thermo Fisher Scientific) with 0.25% BSA, epithelial crypts were extracted. After passing the suspension through a 70 μm cell strainer, crypt concentration was quantified by eye. To achieve a density of 250–750 crypts per dome, crypts were centrifuged at 300 × *g* for 10 min, resuspended in DMEM F12 containing 75% Matrigel (Corning, Corning, NY, USA), and seeded in 50 μL domes in multiwell plates (Sarstedt AG & Co. KG, Nümbrecht, Germany). After 10 min of solidification at 37°C, organoid growth medium (OGM, IntestiCult Human organoid growth medium, STEMCELL technologies) supplemented with 10 μM ROCK Inhibitor Y-27632 (STEMCELL Technologies) and 100 U/mL penicillin-streptomycin (Gibco, Thermo Fisher Scientific) was added. After two days, ROCK Inhibitor Y-27632 was removed from cultures. From then on, the medium was replaced every 3 to 4 days. Following establishment, enteroids were expanded by continuous enteroid subculturing at day 7. At passage 1–4, newly formed enteroids were frozen in DMEM F12 supplemented with 10% fetal bovine serum (FBS) and 10% dimethyl sulfoxide and cryopreserved in liquid nitrogen. Enteroids from four different donors (Hu18-9jej, Hu22-2jej, Hu22-3jej and Hu22-5jej) were used.

#### Enteroid expansion, differentiation and harvesting for experiments

Cryopreserved enteroids were thawed and resuspended in a cold mixture containing 75% Matrigel and 25% OGM. Three 15–20 μL domes of the suspended cells were added to each well in a 24-well plate. The domes were solidified for 10 min at 37°C, then overlaid with 500 μL of OGM containing 100 U/mL penicillin-streptomycin (Pen Strep, Gibco, ThermoFisher Scientific) and 10 μM of ROCK inhibitor Y-27632 for 2 days. Afterward, OGM was replenished excluding Y-27632 but containing 100 U/mL penicillin-streptomycin. The medium was replaced with fresh OGM medium every 2 to 3 days and cultures were maintained at 37°C in 5% CO_2_ until enteroids were large enough for expansion. Thereafter, enteroids were passaged every 7 to 10 days. Briefly, the Matrigel domes were dispersed through repeated gentle pipetting with 1 mL of Gentle Cell Dissociation Reagent per well. The enteroids were then centrifuged at 300 × *g* for 5 min at 4°C. The pelleted enteroid fragments were washed with 1 mL per well of DMEM F12 supplemented with 0.25% BSA, centrifuged a second time at 300 × *g* for 5 min at 4°C, diluted by 1:3 to 1:8 and re-embedded in 15–20 μL Matrigel domes for further expansion as described above. Passages 4 to 20 were used for all experiments. Enteroid differentiation was initiated at day 3 post splitting. OGM was removed and 500 μL of cold Cell Recovery Solution (Corning) was added. Matrigel domes were broken by gently pipetting up and down three times with a 1 mL pipette tip. Enteroids were transferred to a 15 mL Falcon tube and incubated on ice for 1 h on a nutating table (20 rpm, FisherBrand, Pittsburg PA, USA). Supernatant was carefully removed and enteroid pellets were washed with 3 mL of ice-cold DMEM F12 + 0.25% BSA. After removing the supernatant, pellets were re-suspended in organoid differentiation medium (ODM, STEMCELL technologies) supplemented with DAPT (STEMCELL technologies). Enteroid suspensions (500 μL per well) were transferred to pre-cooled 24-well ultra-low attachment plates (Corning). Matrigel was added at 7.5% to the medium to maintain the natural basal-out configuration of the enteroids in the suspension cultures. To reverse the polarity of the enteroids in suspension cultures to apical-out, Matrigel was omitted as previously described.^34^ All suspension cultures were maintained at 37°C in 5% CO_2_, and ODM was replaced every second day. Enteroid development was monitored with brightfield microscopy, both in Matrigel domes and in suspension cultures. Prior to all experiments, enteroids were transferred into strainers (25 μm pore size for AO suspension cultures and 40 μm cell strainers for BO suspensions, Funakoshi, Tokyo, Japan) and washed three times with ice-cold DMEM/F12 to remove debris and Matrigel residues.^89^ For global proteomics and immunofluorescence staining, BO enteroids were pre-incubated on ice with ice-cold cell recovery solution for 1 h (Corning), to ensure close to complete Matrigel removal, ice-cold PBS was used instead of DMEM F12 for both AO and BO enteroid washing. To simplify the description of the different growth conditions of leading to different types of enteroids studied in Figure 1, the nomenclature has been simplified as in Table 1. The first letter indicates enteroids grown in Matrigel (M), basal-out (B) or apical-out (A) configuration, the second letter indicates the growth medium (G) or differentiation medium (D), and the addition of 4 or 6 indicates the number of days the enteroids were cultured in the respective condition before use. Once the most differentiated enteroid suspension culture conditions (AD6 and BD6) were identified for further studies, the nomenclature was simplified to AO and BO, respectively.

#### Global proteomics

The initial global proteomics analysis shown in Figure 1 was performed on enteroids from one donor Hu18-9jej to select the best possible conditions for obtaining differentiated enteroids using two different media, two differentiation times and three different set ups [Matrigel-embedded (M) and suspension (A and B) cultures] (Table 1). Once the optimal conditions were obtained, the number of donors was increased and a second set of experiments using a total of four donors (Hu18-9jej, Hu22-2jej, Hu22-3jej, Hu22-5jej) was performed. This second set of proteomics was performed on MD6, BD6 and AD6 enteroids. Other samples included jejunal tissue [*n* = 5 donors (Hu21-7jej, Hu21-14jej, Hu22-2jej, Hu22-3jej, Hu22-5jej)] and enterocytes [*n* = 4 donors (Hu21-7jej, Hu21-14jej, Hu22-3jej, Hu22-5jej)]. Samples were lysed in lysis buffer (100 mM Tris/HCl, Sigma-Aldrich) containing 50 mM DTT and 2% (w/v) sodium dodecyl sulfate (SDS, Sigma-Aldrich) with a pH of 7.8. The samples were then heated (95°C, 5 min) and sonicated (20 pulses of 1s, 20% amplitude) with a Vibra-Cell ultrasonic processor (Sonics & Materials Inc., Connecticut, US). Multienzyme digestion filter aided sample preparation (FASP) was performed using trypsin (Nordic Biolabs AB, Täby, Sweden) and Lys-C endopeptidase (Thermo Fisher Scientific) to digest proteins to peptides.^90^ An amount of 10 μg of peptide mixture was dried using a SpeedVac (Thermo Fisher Scientific) and stored at −150°C. Prior to proteomics analysis, 10 μg of the resulting peptide mixture was dissolved in 10 μL mobile phase A [0.1% formic acid (VWR) in Milli-Q water (Milli-Q lab water purification system, Merck, Darmstadt, Germany)]. Proteomic analysis was performed on an Orbitrap Q Exactive HF mass spectrometer (MS, Thermo Fisher Scientific) which was coupled to a nano-liquid chromatography (nLC) system. The MS was set to a Top-N method (Data Dependent Acquisition). An EASY-spray C18-column (50 cm, 75 μm inner diameter, Thermo Fisher Scientific) was used to separate peptides in an acetonitrile/water gradient (0.1% formic acid) at 300 nL/min flow rate. Proteins were identified using MaxQuant software (version 2.7.5.0)^87^ with UniProtKB/Swiss-Prot reference human and mouse proteome dataset.^91^ Residual Matrigel protein left in the enteroid samples was quantified using the Total Protein Approach (TPA).^92^ Subsequently, unique mouse proteins were removed from the proteome and human protein abundance was quantified by TPA. To ensure high-quality quantitation, only proteins identified with at least three unique + razor peptides were considered to be quantified (limit of quantitation, LOQ). Differential expression analysis (adjusted *p*-value ≤0.05, absolute log_2_fold change ≥1) was conducted using the limma (in a screening process where each sample had two replicates) and DEqMS (for the rest of the proteomic dataset) package in R.^86^^,^^93^ Gene set enrichment analysis (adjusted *p*-value ≤0.05, multiple testing correction using Benjamin-Hochberg method) was performed with clusterProfiler (version 4.10.1) in R.^88^ All listed donors were included in the global proteomics analysis unless otherwise specified. The proteomics data from the mass spectrometry analysis are available in the PRIDE^83^ repository with the dataset identifier PRIDE: PXD067571.

#### Brightfield microscopy

Enteroids were strained and resuspended in DMEM-F12 as described above, in an ultra-low attachment plate. Brightfield imaging was performed at 4x, 10x and 20x magnifications using a Nikon Eclipse TS100 Inverted Routine Microscope with a Nikon DS-Fi2 camera mount (Nikon Corporation, Tokyo, Japan). Enteroid size (cross sectional area, Feret’s diameter) and shape descriptors (circularity) for both AO (*n* = 127) and BO (*n* = 94) were approximated using images at 4x magnification. Individual enteroids were segmented and masked using Fiji (version 1.54j).^85^ Shape descriptors were acquired by measuring the masks of the segmented enteroids.

#### Scanning electron microscopy

Enteroids were fixed at 4°C overnight with 2.5% glutaraldehyde (TAAB Laboratories, Aldermaston, England) in 0.1 M PHEM-buffer [60 mM PIPES (Sigma Aldrich), 25 mM HEPES (4-(2-hydroxyethyl)-1-piperazineethanesulfonic acid (Sigma-Aldrich), 10 mM egtazic acid (Merck), and 4 mM MgCl_2_ × 6H_2_O (Sigma-Aldrich), pH 6.9]. Samples were allowed to adhere to poly-D-lysine coated cover slips (Ted Pella, Redding, CA, USA) overnight. Cover slips were washed and dehydrated with a graded series of ethanol using a Biowave pro+ microwave (Pelco, Ted Pella) and subsequently critically point dried (Leica EM300). Cover slips were then mounted with carbon tape on an aluminum stub and coated with 5 nm of platinum (Quorum Q150T ES, Quorum Technologies, Lewes, UK). The sample morphology was analyzed by field-emission scanning electron microscopy (FESEM; Carl Zeiss Merlin) using the Inlens secondary electron detector at an accelerating voltage of 5 kV and probe current of 120 pA.

#### Transmission electron microscopy

Enteroids were fixed with 2.5% glutaraldehyde (TAAB Laboratories) in 0.1 M sodium cacodylate (Sigma-Aldrich) or PHEM-buffer. Samples were washed in buffer and post stained in 0.8% potassium ferricyanide (Sigma-Aldrich), 1% OsO4 (TAAB Laboratories) in buffer, further stained with 1% tannic acid (EMS, Hatfield, PA) in Milli-Q water and 1% uranyl acetate (TAAB Laboratories) and further dehydrated using a graded series of ethanol. Samples were infiltrated using a graded series of Spurr’s resin (TAAB Laboratories) and finally polymerized overnight in an oven at 65°C. All sample preparation except fixation was done using a Biowave Pro+ processing microwave (Pelco). Ultrathin sections of 70 nm were picked up on formvar coated grids and analyzed using a Talos L120C (FEI, Eindhoven, The Netherlands) operating at 120 kV. Micrographs were acquired with a Ceta 16M CCD camera (FEI, Eindhoven, The Netherlands) using Velox software (version 2.14.1.40, Thermo Fisher Scientific, Eindhoven, The Netherlands).

#### Immunofluorescence staining

The enteroids were first fixed for 30 min at room temperature with a paraformaldehyde solution at pH 7.4, consisting of 2% paraformaldehyde (PFA, Thermo Fisher Scientific), 60 mM dibasic sodium phosphate (Sigma-Aldrich), and 14 mM monobasic sodium phosphate (Sigma-Aldrich). Following fixation, enteroids were washed three times with PBS for 10 min each. Enteroids were then permeabilized with 0.1% saponin (Thermo Fisher Scientific) and blocked with 2% donkey serum (Merck) in PBS, each for 30 min at room temperature. Primary antibodies used included mouse anti-zonula occludens-1 (ZO-1, 1:100, Thermo Fisher Scientific), mouse anti-CYP3A4 (1:200, Thermo Fisher Scientific), rabbit anti-ABCG2/BCRP (1:100, Abcam, Cambridge, UK), mouse anti-ABCB1/Pgp (1:100, Thermo Fisher Scientific), and rabbit anti-SLC27A4/FATP4 (1:100, Thermo Fisher Scientific). These antibodies were prepared in a blocking/permeabilization buffer containing 3% donkey serum, 1% saponin, and 1% Triton X-100 (Sigma-Aldrich) at the specified concentrations. Enteroids were resuspended in 150 μL of the primary antibody staining solution and incubated overnight at 4°C in anti-adherence solution (STEMCELL technologies, Vancouver, BC, Canada) pre-treated microcentrifuge tubes (Eppendorf, Hamburg, Germany). Following incubation, enteroids were washed three times with PBS for 10 min each. To detect the proteins of interest, enteroids were incubated with goat anti-rabbit IgG secondary antibody coupled to Alexa Fluor 488 (1:500, Thermo Fisher), goat anti-mouse IgG secondary antibody coupled to Alexa Fluor 488 (1:500, Abcam), or donkey anti-rabbit IgG secondary antibody coupled to Alexa Fluor 568 (4 μg/mL, Thermo Fisher Scientific). Phalloidin conjugated to Alexa Fluor 660 (1:400, Thermo Fisher Scientific) was also added to the secondary antibody solution to stain F-actin filaments. Enteroids were resuspended in 150 μL of secondary antibody solution and incubated overnight at 4°C. After three washes with PBS, each for 10 min, the enteroids were resuspended in 10 μL of PBS and transferred to a microscopy glass slide (KEBO-Lab, Stockholm, Sweden). Excess PBS was removed, and VECTASHIELD Vibrance Antifade Mounting Medium with DAPI (BioNordika, Solna, Sweden) was added to the sample. A coverslip, with vacuum grease applied at the corners as spacers, was placed on top of the slide. The enteroids were imaged using a Zeiss LSM800 confocal microscope equipped with a 20x objective and a 40x immersion oil objective.

#### Live-cell imaging of enteroids in suspension

Live-cell imaging was performed on a custom-built microscope based on an Eclipse Ti2 body (Nikon), using a 10x/0.45 Plan Apo Lambda air objective (Nikon), and a back-lit sCMOS camera with a physical pixel size of 11 μm (Prime 95B, Photometrics), and a final image pixel size of 1.12 μm.^43^ The imaging chamber was maintained at 37°C, 5% CO_2_ in a moisturized atmosphere. Brightfield images were acquired by differential interference contrast (DIC), and fluorescence imaging by the excitation light engine Spectra-X (Lumencor) and emission collection through quadruple band pass filters (89402 & 89403; Chroma) and a spinning disk module (X-light V2, Crest optics).

#### Lucifer yellow barrier integrity assay in live enteroids

Enteroids were washed three times with DMEM F12 and resuspended in DMEM F12 containing 100 μM of LY dilithium salt (Ex/Em 428/536 nm) fluorescent dye (Biotium). The enteroids were incubated at 37°C for 30 min with gentle shaking at 100 rpm (dye loading). The apical-out enteroid suspension was kept in the dye solution throughout the experiment, while the basal-out enteroid suspension was washed three times with DMEM F12 before analysis. Samples were observed 30, 60 and 90 min after addition of the dye at 37°C and 5% CO_2_ using live-cell time-lapse microscopy. In the control experiments, the enteroids were permeabilized by addition of EDTA at a final concentration of 2 mM at 30 min post dye loading. Quantification of LY fluorescence was performed using Fiji (version 1.54j). Integrity in apical-out was determined by measuring the mean gray value of whole enteroids within the same focal plane compared to the mean gray value of the local background (*n* = 44 per time-point). For basal-out, enteroids which had a fluorescent lumen from the start were selected and the mean gray value of the lumen was compared with the local background (*n* = 45 per time-point).

#### Fatty acid uptake assay using live-cell microscopy

Enteroids were washed thrice with warm DMEM F12, resuspended in DMEM F12 containing 2.5 μM C1-BODIPY-C12 (Ex/Em 500/510 nm, Thermo Fisher Scientific) and 5 μM BSA and incubated for 10 min at 37°C.^19^ Then, the enteroids were washed and resuspended in DMEM F12 with HCS LipidTOX Deep Red Neutral Lipid Stain (1:5000, Ex/Em 637/655 nm, Thermo Fisher Scientific).^94^ Live-cell imaging was performed at 60x magnification using the custom-built microscope described above (37°C, 5% CO_2_). Enteroids were imaged at 60x to visualize accumulation of C1-BODIPY-C12 within lipid droplets.

#### Analysis of Pgp function using rhodamine 123 in live enteroids

Enteroids were strained and incubated in DMEM F12 with 10 μM of the Pgp fluorescent substrate rhodamine 123 (Rho123, Ex/Em 508/528 nm, Sigma-Aldrich) in a shaking water bath at 100 rpm for 1 h in 37°C in dark conditions. In control experiments, the enteroids were pre-treated with 2 μM of Pgp inhibitor ELC (Sigma-Aldrich) for 1 h at 37 °C, and subsequently treated with 10 μM of Rho123 in presence of 2 μM ELC in DMEM F12 for 1 h at 37°C. After incubation, the enteroids were washed three times at 37°C, resuspended in warm DMEM F12 and subsequently live imaged at 4x and 60x magnifications using the custom-built microscope described above. Dye localization in each enteroid was determined in 60x magnification images, using the Line Profile function of Fiji: a line was drawn on the brightfield channel across each enteroid’s diameter and the mean gray value in the fluorescent channel was measured along the length of that line. On a population scale, fluorescence was determined by measuring mean gray values in 4x magnification images, using an in-house developed algorithm for Fiji. Herein, the whole AO population was imaged, whereas in basal-out enteroids a preselection was made at the brightfield channel. Only basal-out enteroids exhibiting a differentiated phenotype with budding and a thick epithelium were analyzed. Moreover, the focal plane of basal-out enteroids selected for analysis was strictly aligned with a fully defined lumen. For apical-out enteroids, which have the Pgp transporters facing outwards, whole-enteroid fluorescence was compared to that of the background fluorescence. For basal-out enteroids, which in contrast to apical-out enteroids have a clearly distinguishable lumen, luminal fluorescence of each enteroid was compared to the fluorescence of the surrounding epithelium. Data analysis was performed using GraphPad Prism (version 10). For apical-out enteroids, the mean gray value of Rho123 exposed enteroids (*n* = 102) was compared to that of the control group [Rho123 exposed enteroids pre-incubated with the specific Pgp inhibitor ELC (*n* = 143)].

#### Screening of BCRP efflux transporter activity using pheophorbide A

BCRP activity was measured on AO enteroids using BCRP substrate pheophorbide A (PhA) as previously described.^46^ The MDCK^cMDR1−KO^ (hereafter referred to as MDCK)^75^ at passages 16 to 23 and MDCK-hBCRP^cMDR1−KO^ (hereafter referred to as MDCK-hBCRP)^46^ clones at passages 17 to 24 were used as non-effluxing negative control and BCRP overexpressing positive control respectively. MDCK cells were grown in DMEM Glutamax (Gibco, Thermo Fisher Scientific) supplemented with 10% FBS (Gibco, Thermo Fisher Scientific) and 1% Pen Strep (Sigma-Aldrich). For the MDCK-hBCRP clone, 0.6 mg/mL of geneticin (Gibco, Thermo Fisher Scientific) was additionally used as a selection antibiotic. The cells were passaged twice per week 1 × 10^6^ cells in a T75 flask (Corning).^46^ For the efflux assay, AO enteroids were grown and differentiated as described above. MDCK and MDCK-hBCRP cells were seeded 100.000 cells/well on a 96-well clear bottom plate (Corning) and differentiated for 5 days. The MDCK-hBCRP clone was induced 24 h prior dye addition with 10 mM sterile-filtered sodium butyrate (NaBut) (Merck). All media used for this clone thereafter contained NaBut to maintain induction. On the 5^th^ day of differentiation for AO, MDCK and MDCK-hBCRP, PhA was added in their media. In brief, apical-out enteroids (∼5 × 10^5^ cells) were resuspended in ODM with 1 μM PhA, with or without 2 μM of BCRP inhibitor KO143 and transferred to the Corning 96-well plate. MDCK and MDCK-hBCRP cells were correspondingly kept in their respective growth media with 1 μM PhA with or without 2 μM KO143. After incubation for 18 h, on differentiation day 6, enteroids and both MDCK clones were washed five times with HBSS pH 7.4. Thereafter, relative fluorescence was measured using a Tecan plate reader (Tecan, Grödig, Austria) using excitation and emission wavelengths of 395 nm and 670 nm, respectively. All results were normalized to number of cells per sample as counted using a NucleoCounter NC-100 (ChemoMetec A/S, Allerød, Denmark). Results were visualized as PhA accumulation with KO143 over the accumulation without inhibitor.

#### CYP3A4 metabolic activity

Enteroids were resuspended at a density corresponding to 3 × 10^6^ enterocytes per well (NucleoCounter, ChemoMetec A/S) and incubated with 1 μM of either of the CYP3A4 substrates terfenadine or midazolam (Sigma-Aldrich) for 2 h. Enteroids from donor Hu18-9jej and enterocytes from three donors (Hu22-32jej, Hu23-01jej and Hu23-05jej) were used. As negative control, cells were heat-inactivated for 5 min at 95°C and cooled on ice before the experiment. Cell suspensions and compound solutions were equilibrated to 37°C prior to experiment start. Samples were collected at 0, 30, 60 and 120 min and lysed with 120 μL 100% acetonitrile containing 5 nM warfarin as internal standard. Samples were centrifuged at 4°C at 3500 rpm for 20 min. A sample volume of 100 μL was transferred to a new plate and 100 μL of 60% acetonitrile containing 3 nM warfarin was added. The protein content of the pellets was quantified using the tryptophan method.^95^ Compound concentrations were determined by UPLC-MS/MS using an Acquity UPLC coupled to a XEVO TQ triple-quadrupole mass spectrometer with positive electrospray ionization (Waters Corp., Milford, MA). For chromatographic separation, a C18 BEH 1.7 μm column (50 mm, Waters Corp.) was used, with a general gradient of 1%–90% of mobile phase B over a total running time of 5 min. Mobile phase A consisted of 5% acetonitrile and 0.1% formic acid in purified water, and mobile phase B of 0.1% formic acid in 100% acetonitrile. The flow rate was set to 0.5 mL/min and 5 μL of the sample was injected. Multiple reaction monitoring methods were optimized for each compound, metabolite and internal standard. The intrinsic clearance (CL_int_) was calculated according to:

(Equation 1) $${CL}_{\mathrm{int}}=\left[\;\mu L\times \min^{-1}\times {\left(1\times 10^{6}\;cell\right)}^{-1}\right]=\frac{V\times \ln\left(2\right)}{t_{1/2}}$$

where V indicates the volume of incubation (μL) and t_**1/2**_ refers to the compound half-life.

#### Physiologically-based pharmacokinetic simulation

Physiologically-based pharmacokinetic (PBPK) simulations were performed using the Simcyp simulator (V23 r2, Certara Predictive Technologies, Sheffield, United Kingdom). For simulations using AO enteroid data, the intestinal metabolism in the baseline model was replaced with the *in vitro* CL_int_ measured in AO enteroids. The CL_int_ ((μL × min^−1^ × (1×10^6^ cells)^−1^) was converted to metabolic clearance per mg of microsomal protein by correcting for the measured protein abundance per million cells. It was assumed that the endoplasmic reticulum accounts for 40% of total cellular protein. The *in vitro* CL_int_ was corrected for binding,^96^ assuming similar binding characteristics between intestinal and hepatic cells. The final CL_int_ applied in the model was 177.6 μL/min/mg microsomal protein in the intestine (coefficient of variation = 11%). In control simulations where intestinal metabolism was omitted, the intestinal CL_int_ was set to zero. A PBPK model for terfenadine was constructed using the input parameters listed in Table S13.^97^^,^^98^^,^^99^^,^^100^^,^^101^ Terfenadine absorption was simulated using the advanced dissolution, absorption and metabolism (ADAM) model,^102^ with permeability predicted via a mechanistic permeability model.^103^ A minimal PBPK model described distribution, with peripheral compartment parameters adjusted to match published concentration-time profiles.^104^ Elimination was primarily via CYP3A4 (fraction metabolized: ∼0.98), consistent with those reported earlier.^105^ Intestinal binding [(unbound fraction (fu_gut_)] was set to 0.15, yielding an intestinal availability (F_g_) of ∼0.35 in the baseline model. This value of F_g_ was in line with the value estimated for F_g_ based on the assumption that grapefruit juice inhibits CYP3A in the intestine with minimal effects on hepatic CYP3A4 and aligned with earlier estimates by Gertz et al. (40%).^105^ The same fu_gut_ value was used both for the baseline model and the model using AO enteroid metabolism data. Midazolam simulations used the default midazolam file within the Simcyp simulator, simulating a 2 mg oral dose (10 trials of 15 subjects, 18–55 years, 19% female). Clinical data were sourced from the literature.^106^^,^^107^^,^^108^^,^^109^

### Quantification and statistical analysis

LY data were analyzed with Kruskal-Wallis test and multiple comparison analysis. Significant differences between the Rho123 exposed enteroids and the control group were investigated using Mann-Whitney U test. For basal-out enteroids, Wilcoxon’s signed rank test was used for both Rho123 treated (*n* = 154) and Rho123 + ELC treated (*n* = 102) populations, since each enteroid’s luminal gray value was compared to its epithelial gray value. Statistical analysis and figures were made using GraphPad Prism (version 10). Specific statistical details of each experiment are listed in the corresponding figure legends.

## Supplemental information

Document S1. Figures S1–S6 and Tables S1–S5 and S7–S13

Table S6. SLC transporter protein abundance in AO and BO enteroids, enterocytes and jejunal mucosa
