# Supplementary material for: Proteomic and functional profiling of suspended jejunal enteroids reveals pharmacokinetic fidelity to native intestine
Source: iScience. 2026 Sep 30;29(10):117654. doi: 10.1016/j.isci.2026.117654 (PMC13657221; doi:10.1016/j.isci.2026.117654)
Supplement: Supplementary file 1 [file mmc1.pdf]

iScience, Volume *29*

Supplemental information

Proteomic and functional profiling of suspended jejunal enteroids reveals pharmacokinetic fidelity to native intestine

Foteini Tzioufa, Merve Ceylan, Maria Letizia Di Martino, Dinh Son Vo, Ana C.C. Lopes, Rebekkah Hammar, Magnus Sundbom, Martin Skogar, Per M. Hellström, Dominic-Luc Webb, Maria Karlgren, Iain Gardner, Patrik Lundquist, Jens Eriksson, Daisy Hjelmqvist, Mikael E. Sellin, Madlen Hubert, and Per Artursson

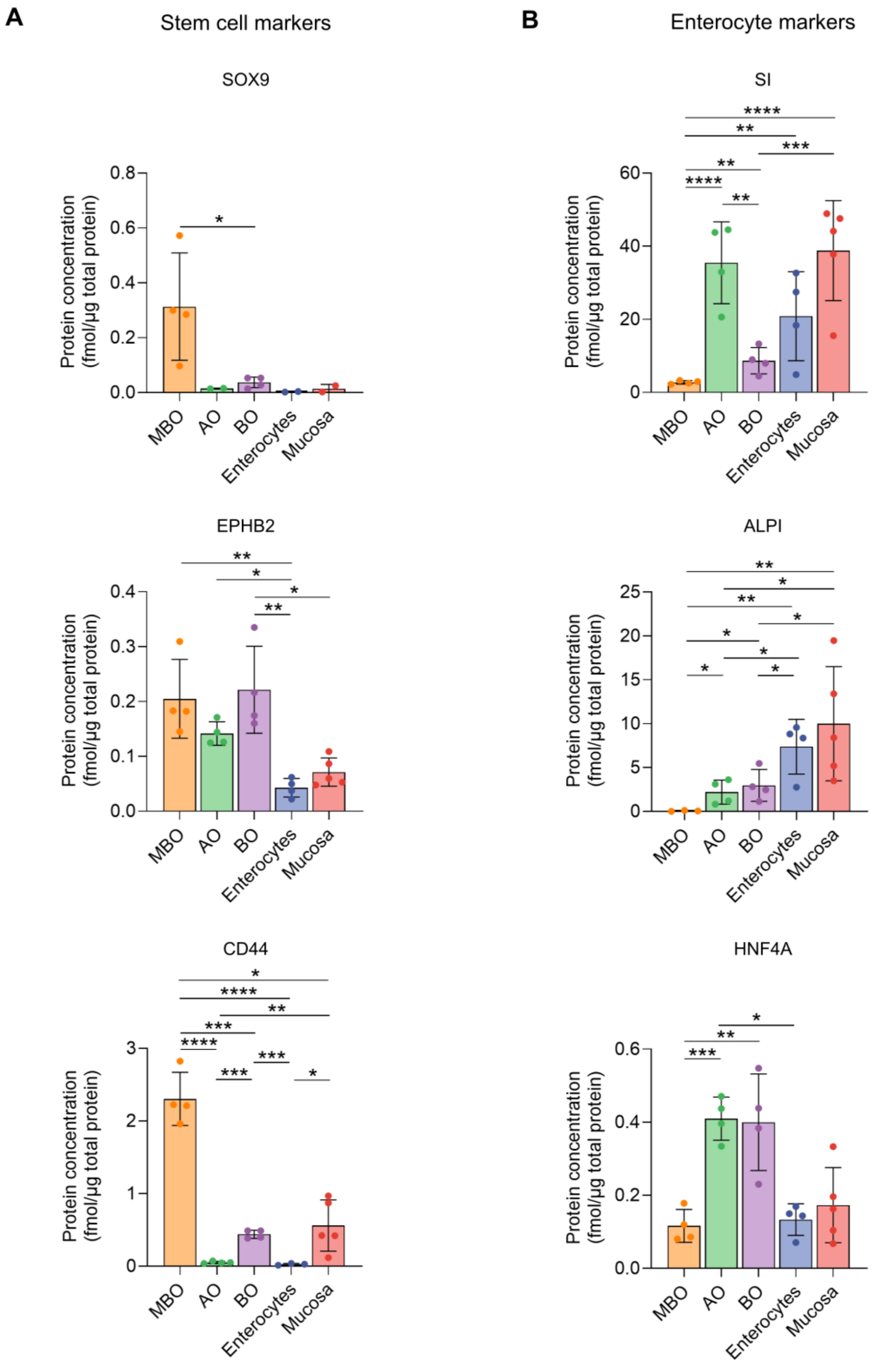

Figure S1. Global protein profiling of human enteroids in various culture formats, Related to Figure 1. Quantification of **(A)** SOX9, EPHB2, CD44 (stem cell and proliferative markers, left) and **(B)** SI, ALPI, HNF4A (enterocyte markers, right) across 3D enteroid formats (MBO, AO and BO) cultured in ODM for 6 days, compared to primary enterocyte and jejunal mucosa *in vivo* levels. Data from different donors (enteroids, n = 4: Hu22-2jej, Hu22-3jej, Hu22-5jej, Hu18-9jej; enterocytes, n = 4: Hu22-3jej, Hu22-5jej, Hu21-7jej, Hu21-14jej; jejunal mucosa, n = 5: Hu22-2jej, Hu22-3jej, Hu22-5jej, Hu21-7jej, Hu21-14jej) are shown as mean ± SD. *$p < 0.05$, **$p < 0.01$, ***$p < 0.001$, ****$p < 0.0001$ (DEqMS). measurements using brightfield images.

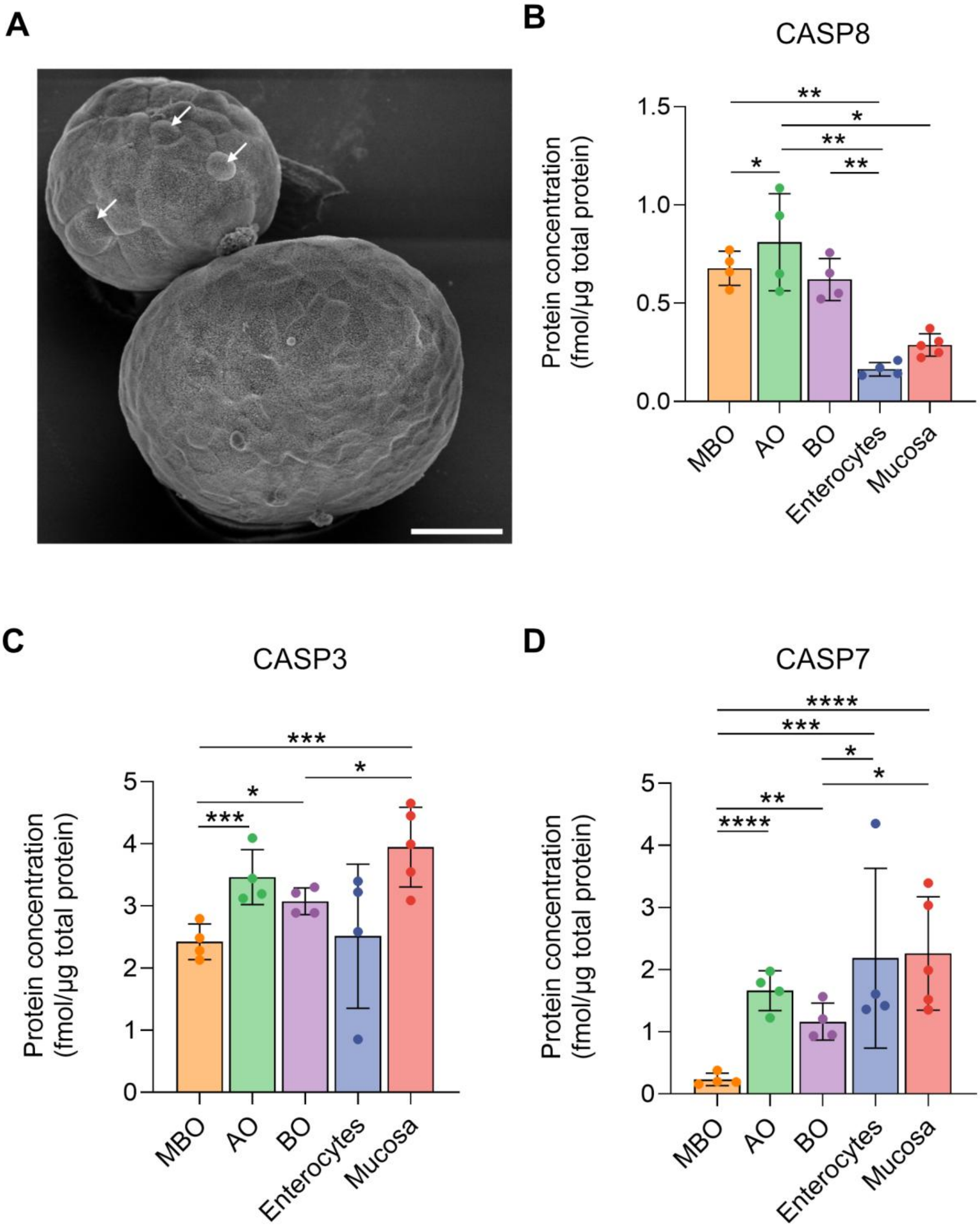

Figure S2. Morphology of cell extrusion from apical-out enteroids and expression of apoptosis-related proteins, Related to Figure 3. (A) SEM image of apical-out (AO) enteroids where single enterocytes are beginning to bulge from enterocyte monolayer (as highlighted by white arrows). Scale bars, 20 µm. **(B-D)** Concentrations of apoptosis related proteins (CASP8, CASP3, CASP7 present in different 3D enteroid formats cultured in ODM for 6 days (MBO, AO and BO) was compared to primary enterocyte and jejunal mucosa *in vivo* levels. Data from different donors (enteroids, n = 4: Hu22-2jej, Hu22-3jej, Hu22-5jej, Hu18-9jej; enterocytes, n = 4: Hu22-3jej, Hu22-5jej, Hu21-7jej, Hu21-14jej; jejunal mucosa, n = 5: Hu22-2jej, Hu22-3jej, Hu22-5jej, Hu21-7jej, Hu21-14jej) are shown as mean ± SD. *$p < 0.05$, **$p < 0.01$, ***$p < 0.001$, ****$p < 0.0001$ (DEqMS).

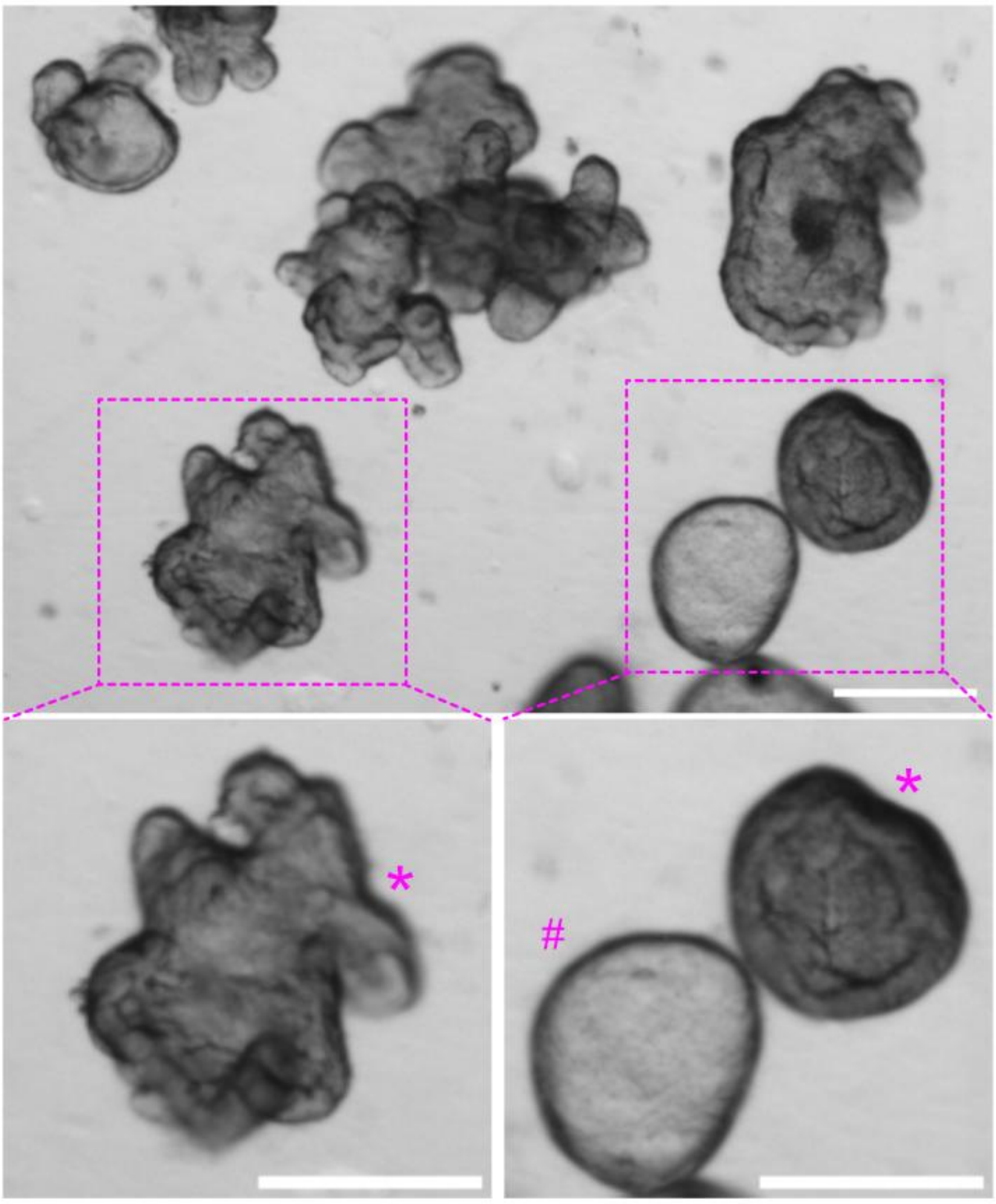

Figure S3. Morphology of differentiated 3D basal-out enteroids in suspension, Related to Figure 2. Representative DIC micrographs of live BO enteroids (donor Hu18-9jej) at day 6 of differentiation in suspension. The image illustrates fully differentiated enteroids (marked by *) and smaller enteroids with a stem-like phenotype (marked by #). Scale bars, 200 µm.

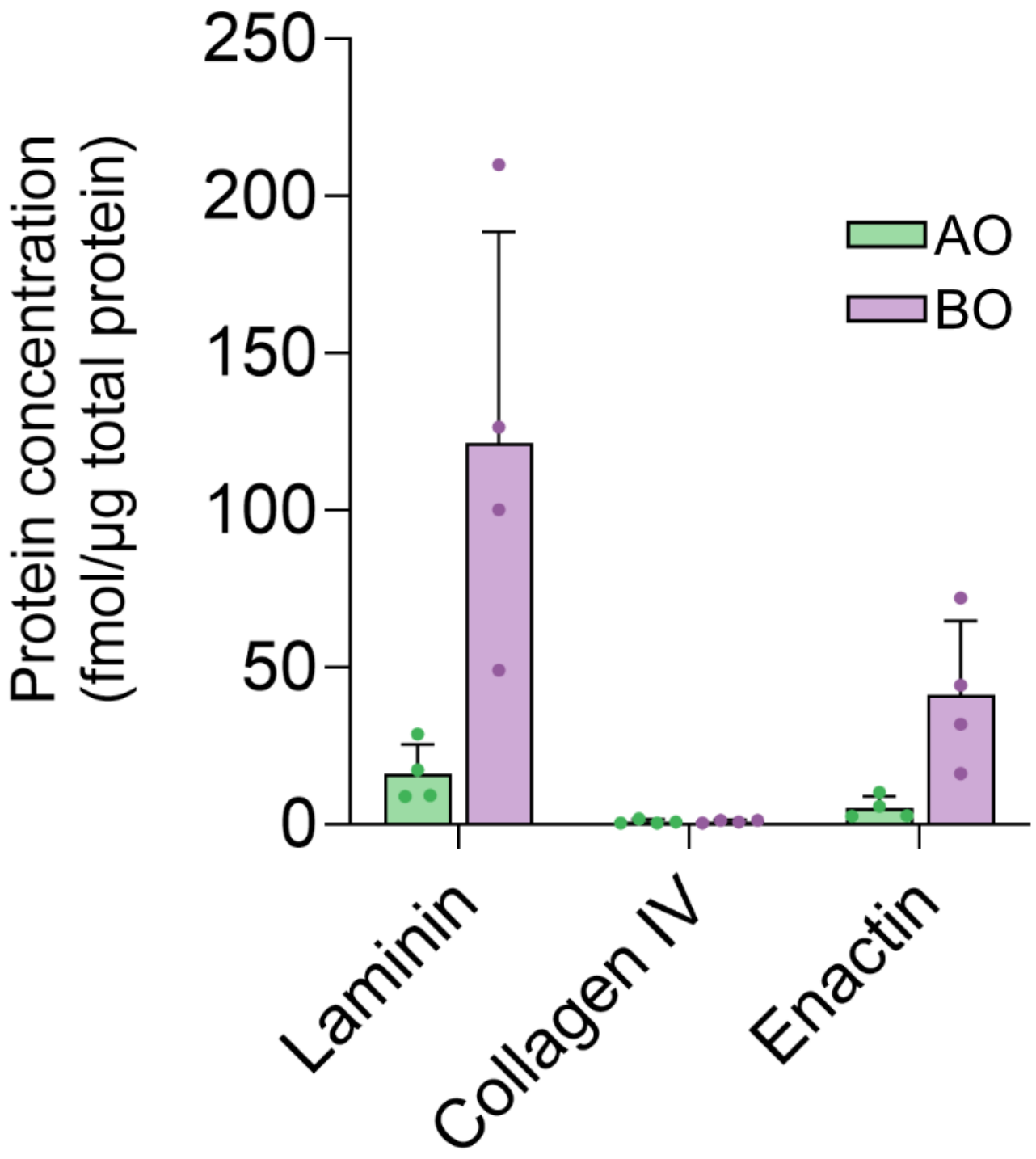

Figure S4. Concentrations of Matrigel proteins in AO and BO enteroids in suspension culture, **Related to Figure 2.** Data from different donors (Hu22-2jej, Hu22-3jej, Hu22-5jej, Hu18-9jej) are shown as mean ± SD.

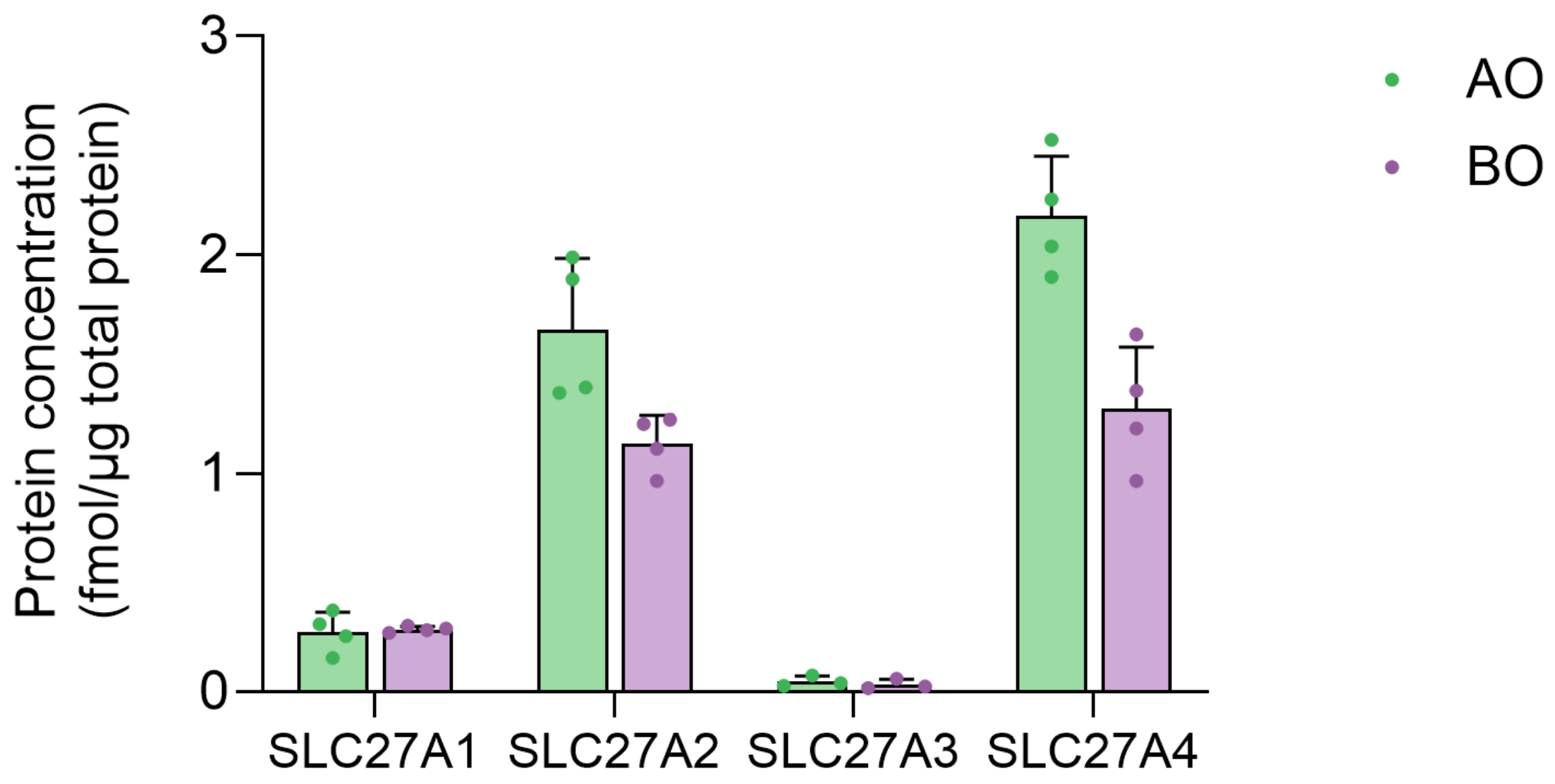

Figure S5. Expression of fatty acid transporting proteins (FATPs), Related to Figure 5. Expression levels of FATPs as determined by global proteomics in AO and BO enteroids. Data from different donors (Hu18-9jej, Hu22-2jej, Hu22-3jej, Hu22-5jej) are shown as mean ± SD.

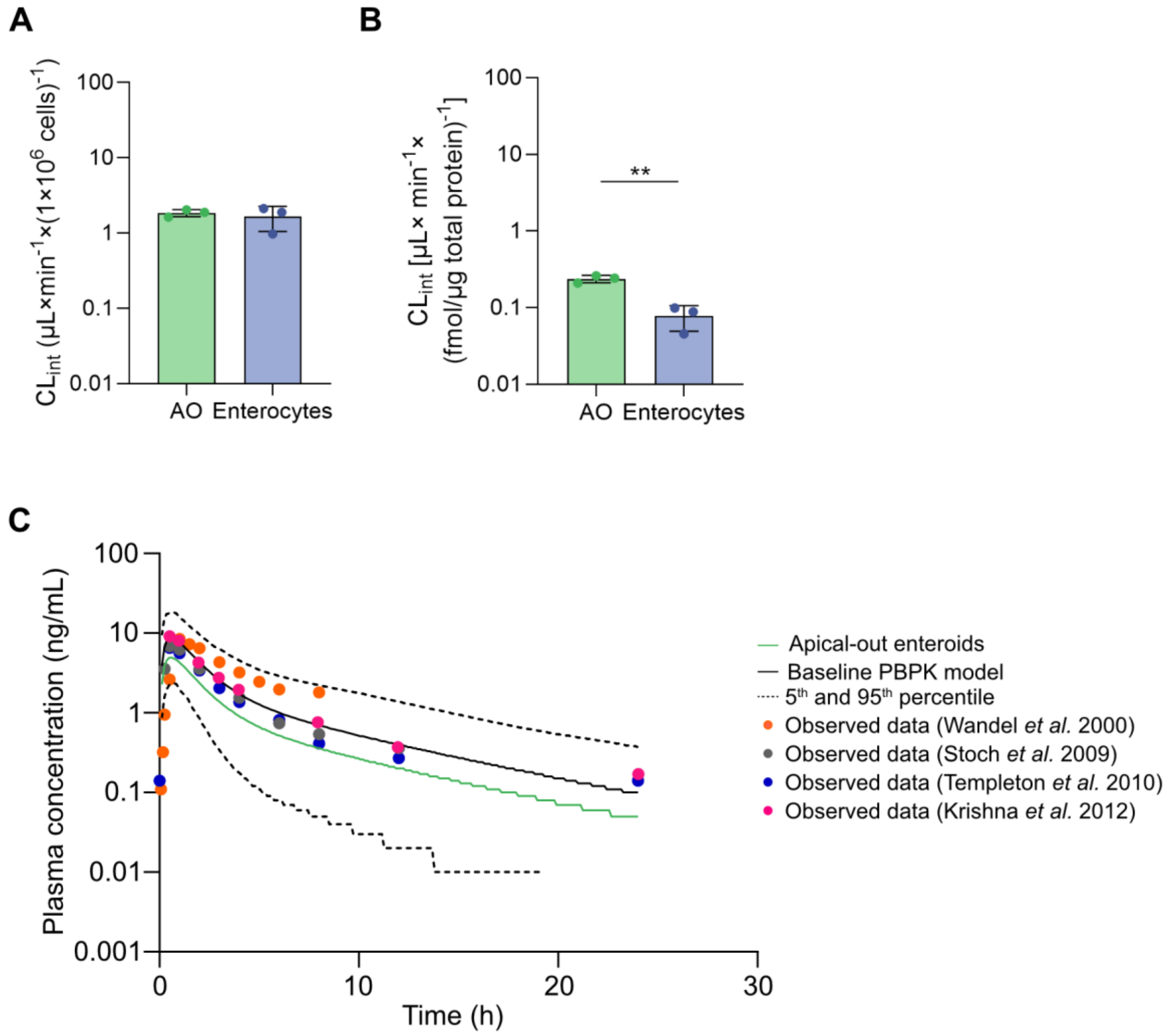

Figure S6. CYP3A4 metabolism of midazolam by AO enteroids and PBPK modelling of midazolam pharmacokinetics, Related to Figure 8. (A) Intrinsic clearance of midazolam in apical-out enteroids (donor Hu18-9jej) and primary enterocytes (donors: Hu22-32jej, Hu23-01jej and Hu23-05jej). Data is shown as mean ± SD. Non-significant change, unpaired t-test **(B)** Corrected intrinsic clearance of midazolam considering the differences in CYP3A4 protein expression in apical-out enteroids (donor Hu18-9jej) and primary enterocytes (donors: Hu22-32jej, Hu23-01jej and Hu23-05jej). Data is shown as mean ± SD; $^{**}p < 0.01$, unpaired t-test. **(C)** Simulated pharmacokinetics of a 5 mg dose of midazolam given to 100 virtual subjects. The green line represents the PBPK model mean prediction using the apical-out organoids to describe the rate of intestinal metabolism (log concentration). The black line represents mean predicted plasma concentration for the baseline midazolam model within the Simcyp simulator. The metabolism in the liver and intestine in this model is scaled from *in vitro* data in recombinant CYP3A4 and 5 supersomes. The dashed lines are the 5th and 95th percentiles of the population predicted using the baseline model. The colored dots depict mean observed data from the clinical studies of Wandel *et al*.[S1], Stoch *et al*.[S2], Templeton *et al*.[S3] and Krishna *et al*.[S4]

Table S1. Group level demographic information on the donor tissue samples used in this study, Related to STAR Methods. The age groups included young adult (< 31), middle-age adult (31-45) and old-aged adults (> 46).

**Age group**	**No**	**% Female**
Young adult	1	100%
Middle -age adult	5	80%
Old-age adult	3	67%
Total	9	
Average age	42	

Table S2. Summary of size and shape parameters for AO and BO enteroids, Related to Figure 2. Analysis is based on measurements conducted on brightfield and SEM images. Data are presented as mean ± SD or median (Q1-Q3) and n indicates number of enteroids analyzed. Enteroids from donor Hu18-9jej were used.

**Parameter**	**AO enteroids**	**BO enteroids**[§§]
Mean enterocyte height (μm)[*]	29.8 ± 11.6<br>(n = 59)	-
Mean enterocyte width (μm)[§]	11.27 ± 3.20<br>(n = 19)	-
Median cross-sectional area (μm^2)[#]	12875<br>(6942-18971)<br>(n = 122)	26436<br>(14573-50205)<br>(n = 94)
Median circularity[#]	0.86<br>(0.80-0.88)<br>(n = 118)	0.54<br>(0.47-0.65)<br>(n = 94)
Mean enteroid diameter (μm)[##]	139.9 ± 47.33<br>(n = 118)	233.2 ± 118.9<br>(n=94)
Mean enteroid radius (μm)[$]	75.13 ± 28.17<br>(n = 19)	
Mean approximate cell number[**]<br>(mean enteroid surface area/mean enterocyte apical surface area)	711.19 ± 115.21<br>(n = 19)	-

[*]Mean cell height was calculated by drawing a line across the axis of AO enterocytes in 20x magnified brightfield images. Data presented as mean ± SD.
[§]Mean cell width was calculated by drawing a line across the apical side of AO enterocytes in 20x magnified brightfield images. Data presented as mean ± SD.
[#]Median enteroid cross-sectional areas and Feret diameters were approximated as described at the materials and methods section; Data presented as median (Q1-Q3) where Q1 is the 25% and Q3 is the 75% percentile of the IQR; brightfield microscopy.
[##]Mean enteroid diameters were approximated as described at the materials and methods section; Data presented as mean ± SD; brightfield microscopy
[$]Mean enteroid radius was approximated by dividing the average feret diameter of 19 randomly selected enteroids estimated in [#] by 2. Calculations were performed with Microsoft Office Excel and GraphPad Prism 10.
[**]Cell number per enteroid was calculated by assuming that the enteroid population comprises perfectly spherical enteroids and that the numerical value of the hexagonal apical surface area is the same as that of a circle. As such the sphere surface area (A_S) equation (1) was implemented to find the median enteroid apical surface area:

$$(1)\ \mathrm{As} = 4\pi r_0^2$$

Where r_0 is the mean radius calculated for n = 19 randomly selected enteroids. This resulted in a mean surface area of A_s = 70945.76 μm^2.

For the mean apical enterocyte width, the circle area (A_A) equation (2) was utilized:

$$(2)\ A_A = \pi r_e^2$$

Where r_e is the mean radius of the apical enterocyte surface width and was calculated by dividing the values in [§] by 2. The mean surface area for the apical side of the enterocytes was found to be 99.7 μm^2.

Median enterocyte number was approximated by dividing

$$(1)/(2) = A_S / A_A \approx 711$$

§§Due to the apical side being contained inside the enteroids, it was not possible to acquire enterocyte

Table S3. Abundance of proteins related to microvillus structure in human enteroids, primary enterocytes, and jejunal mucosa, Related to Figure 2. Protein levels are given in fmol/µg total protein. Data are presented as mean (min – max) and n indicates the number of donors for which the protein was quantified. Protein concentration in different 3D enteroid formats cultured in ODM for 6 days (MBO, AO and BO) was compared to primary enterocyte and jejunal mucosa *in vivo* levels. Data from different donors (enteroids: Hu22-2jej, Hu22-3jej, Hu22-5jej, Hu18-9jej; enterocytes: Hu22-3jej, Hu22-5jej, Hu21-7jej, Hu21-14jej; jejunal mucosa: Hu22-2jej, Hu22-3jej, Hu22-5jej, Hu21-7jej, Hu21-14jej) are shown as mean. ND (not detected) indicates proteins that were not detected, and NQ (not quantified) refers to proteins that were identified but had expression levels below the limit of quantification.

**Protein**	**MBO**	**AO**	**BO**	**Enterocytes**	**Mucosa**
	Mean<br>(min - max)<br>n	Mean<br>(min - max)<br>n	Mean<br>(min - max)<br>n	Mean<br>(min - max)<br>n	Mean<br>(min - max)<br>n
ANKS4B	0.09<br>(0.08–0.10)<br>3	0.62<br>(0.39–1.13)<br>4	0.53<br>(0.33–0.76)<br>4	1.41<br>(1.17–1.56)<br>4	1.01<br>(0.87–1.12)<br>4
CDHR2	0.02<br>(0.02–0.02)<br>2	0.83<br>(0.49–1.23)<br>4	0.47<br>(0.21–0.77)<br>4	0.10<br>(0.08–0.16)<br>4	1.78<br>(0.44–5.10)<br>5
CDHR5	NQ	2.76<br>(2.18–3.02)<br>4	0.65<br>(0.49–0.77)<br>4	0.78<br>(0.60–0.91)<br>3	1.33<br>(0.92–2.21)<br>4
ESPN	0.32<br>(0.10–0.43)<br>4	0.47<br>(0.35–0.59)<br>4	0.33<br>(0.30–0.36)<br>4	0.90<br>(0.28–1.50)<br>4	0.48<br>(0.22–0.80)<br>4
EPS8	0.76<br>(0.62–0.87)<br>4	1.21<br>(1.11–1.36)<br>4	1.42<br>(1.26–1.65)<br>4	0.92<br>(0.59–1.30)<br>4	0.80<br>(0.31–1.03)<br>5
EZR	27.55<br>(24.00–30.38)<br>4	50.19<br>(49.51–50.86)<br>4	34.58<br>(29.48–39.20)<br>4	38.74<br>(19.25–54.39)<br>4	39.12<br>(29.28–48.93)<br>5
FUT2	0.46<br>(0.35–0.57)<br>2	1.18<br>(0.92–1.44)<br>2	2.11<br>(1.67–2.55)<br>2	0.66<br>(0.66–0.66)<br>1	0.66<br>(0.35–0.97)<br>2
FUT3	1.53<br>(0.38–2.73)<br>4	1.24<br>(0.26–2.31)<br>4	2.88<br>(0.66–5.24)<br>4	1.29<br>(0.47–2.10)<br>4	1.09<br>(0.22–1.72)<br>5
FUT8	0.38<br>(0.28–0.45)<br>4	0.17<br>(0.11–0.23)<br>4	0.26<br>(0.11–0.39)<br>4	0.10<br>(0.07–0.13)<br>2	0.09<br>(0.05–0.12)<br>3
PDZK1	0.10<br>(0.04–0.14)<br>3	6.62<br>(5.36–8.01)<br>4	1.15<br>(0.55–1.78)<br>4	4.50<br>(3.10–6.23)<br>4	4.39<br>(2.28–5.42)<br>5
MUC1	0.93<br>(0.36–1.71)<br>4	1.09<br>(0.50–1.68)<br>4	1.98<br>(0.55–3.97)<br>4	NQ	NQ
MUC13	0.39<br>(0.28–0.61)<br>4	8.64<br>(5.74–12.21)<br>4	8.99<br>(3.80–20.13)<br>4	6.57<br>(4.86–8.31)<br>4	6.44<br>(3.10–8.79)<br>5

MUC17	0.02 (0.02–0.03) 4	0.07 (0.03–0.09) 4	0.08 (0.07–0.10) 4	0.01 (0.01–0.02) 2	0.01 (0.01–0.03) 3
MYO1A	0.28 (0.23–0.33) 4	4.49 (2.84–6.50) 4	2.39 (2.10–2.88) 4	14.49 (8.57–26.08) 4	13.89 (11.42–18.19) 5
VIL1	36.25 (33.78–39.79) 4	57.47 (51.60–68.20) 4	44.10 (39.32–46.75) 4	61.87 (33.26–90.70) 4	58.70 (43.58–73.21) 5

Table S4. Abundance of tight junction, adherens junction and desmosomal proteins in human 3D enteroids, primary enterocytes, and jejunal mucosa, Related to Figure 3. Protein levels (fmol/µg total protein) in different 3D enteroid formats cultured in ODM for 6 days (MBO, AO and BO) was compared to primary enterocyte and jejunal mucosa *in vivo* levels. Data from different donors (enteroids: Hu22-2jej, Hu22-3jej, Hu22-5jej, Hu18-9jej; enterocytes: Hu22-3jej, Hu22-5jej, Hu21-7jej, Hu21-14jej; jejunal mucosa: Hu22-2jej, Hu22-3jej, Hu22-5jej, Hu21-7jej, Hu21-14jej) are presented as mean (min – max) and n indicates the number of donors for which the protein was quantified. ND (not detected) indicates proteins that were not detected, and NQ (not quantified) refers to proteins that are identified but have expression levels below the limit of quantification.

Protein	MBO	AO	BO	Enterocytes	Mucosa
	Mean<br>(min - max)<br>n	Mean<br>(min - max)<br>n	Mean<br>(min - max)<br>n	Mean<br>(min - max)<br>n	Mean<br>(min - max)<br>n
CDH1	2.96<br>(2.62–3.26)<br>4	4.54<br>(4.06–5.04)<br>4	4.19<br>(3.95–4.54)<br>4	2.58<br>(1.77–3.13)<br>4	4.08<br>(1.81–8.66)<br>5
CGN	0.86<br>(0.79–0.92)<br>4	2.41<br>(2.12–2.57)<br>4	1.00<br>(0.85–1.26)<br>4	2.83<br>(2.01–3.73)<br>4	1.86<br>(0.98–2.33)<br>5
CLDN2	2.04<br>(1.38–2.82)<br>4	NQ	0.40<br>(0.40–0.40)<br>1	NQ	ND
CLDN3	2.66<br>(2.16–3.11)<br>4	5.25<br>(4.91–5.60)<br>4	4.96<br>(3.79–5.90)<br>4	1.69<br>(1.50–1.88)<br>2	2.65<br>(2.05–3.01)<br>3
CLDN7	1.36<br>(1.36–1.36)<br>1	1.81<br>(1.81–1.82)<br>2	2.52<br>(2.25–2.79)<br>2	2.03<br>(1.24–3.35)<br>3	0.88<br>(0.88–0.88)<br>1
CLDN15	0.35<br>(0.32–0.37)<br>2	0.99<br>(0.85–1.10)<br>4	1.28<br>(0.90–1.72)<br>4	0.65<br>(0.55–0.74)<br>2	0.48<br>(0.48–0.48)<br>1
CTNND1	4.96<br>(4.65–5.50)<br>4	5.96<br>(4.91–7.07)<br>4	6.07<br>(5.48–6.66)<br>4	7.36<br>(5.82–10.22)<br>4	7.84<br>(5.02–13.98)<br>5
DSG2	6.52<br>(5.79–7.19)<br>4	1.70<br>(0.82–2.27)<br>4	6.14<br>(5.07–6.78)<br>4	2.62<br>(2.11–3.43)<br>4	2.49<br>(1.72–3.18)<br>5
DSC2	0.79<br>(0.56–0.97)<br>4	0.38<br>(0.12–0.77)<br>4	1.41<br>(1.30–1.47)<br>4	0.69<br>(0.54–0.87)<br>4	0.84<br>(0.56–1.05)<br>5
DSP	8.71<br>(7.39–9.59)<br>4	6.13<br>(4.41–6.97)<br>4	8.17<br>(6.87–9.39)<br>4	7.39<br>(5.38–10.86)<br>4	5.22<br>(3.56–6.97)<br>5
MARVELD2	NQ	0.05<br>(0.05–0.05)<br>1	0.07<br>(0.07–0.07)<br>1	NQ	NQ
OCLN	0.34<br>(0.12–0.44)<br>4	0.61<br>(0.40–0.75)<br>4	0.37<br>(0.18–0.47)<br>4	0.45<br>(0.04–1.11)<br>4	0.22<br>(0.17–0.30)<br>3

PKP2	4.42<br>(3.13–6.07)<br>4	2.37<br>(1.44–3.72)<br>4	4.76<br>(4.31–5.11)<br>4	1.09<br>(0.77–1.84)<br>4	1.79<br>(0.86–2.29)<br>5
TJP1	0.77<br>(0.63-0.87)<br>4	1.16<br>(0.90-1.41)<br>4	0.50<br>(0.45-0.55)<br>4	0.38<br>(0.35-0.42)<br>4	0.31<br>(0.18-0.40)<br>5
TJP2	1.34<br>(1.20–1.49)<br>4	1.83<br>(1.67–2.16)<br>4	2.22<br>(2.00–2.46)<br>4	1.24<br>(0.97–1.61)<br>4	0.92<br>(0.74–1.24)<br>5
TJP3	0.54<br>(0.45–0.59)<br>4	1.58<br>(1.15–2.05)<br>4	1.11<br>(1.02–1.19)<br>4	0.34<br>(0.08–0.51)<br>4	0.55<br>(0.38–0.79)<br>5
VCL	10.88<br>(9.25–13.66)<br>4	10.62<br>(8.00–12.00)<br>4	8.69<br>(8.06–9.97)<br>4	4.59<br>(2.79–6.19)<br>4	13.53<br>(8.53–19.12)<br>5

Table S5. Number of quantified ADME transporters and enzymes in human enteroids, primary enterocytes, and jejunal mucosa as determined by global proteomics, Related to Figure 4.

	MBO	AO	BO	Enterocyte	Mucosa	Total
ABCs	18	19	18	19	19	19
SLCs	102	108	110	106	108	110
CYPs	16	16	17	16	16	17
ADHs	5	5	5	6	6	6
ALDHs	15	15	15	15	16	16
CESs	3	3	3	3	3	3
FMOs	3	3	3	3	3	3
UGTs	7	11	11	11	11	11
SULTs	6	7	8	8	8	8
GSTs	14	15	15	13	15	15
NATs	5	5	4	4	4	5
Total proteins	194	207	209	204	209	213

Table S7. Abundance of ABC transporters in human 3D enteroids, primary enterocytes, and jejunal mucosa, Related to Figure 4. Protein levels (fmol/µg total protein) in different 3D enteroid formats cultured in ODM for 6 days (MBO, BO and AO) was compared to primary enterocyte and jejunal mucosa *in vivo* levels. Data from different donors (enteroids: Hu22-2jej, Hu22-3jej, Hu22-5jej, Hu18-9jej; enterocytes: Hu22-3jej, Hu22-5jej, Hu21-7jej, Hu21-14jej; jejunal mucosa: Hu22-2jej, Hu22-3jej, Hu22-5jej, Hu21-7jej, Hu21-14jej) are presented as mean (min – max) and n indicates the number of donors for which the protein was quantified. ND (not detected) indicates proteins that were not detected, and NQ (not quantified) refers to proteins that were identified but had expression levels below the limit of quantification.

Protein	MBO	AO	BO	Enterocytes	Mucosa
	Mean<br>(min - max)<br>n	Mean<br>(min - max)<br>n	Mean<br>(min - max)<br>n	Mean<br>(min - max)<br>n	Mean<br>(min - max)<br>n
ABCA2	NQ	NQ	NQ	NQ	ND
ABCA8	ND	ND	ND	ND	NQ
ABCB1	0.04<br>(0.03–0.09)<br>4	2.16<br>(1.43–2.68)<br>4	1.90<br>(1.08–2.89)<br>4	0.82<br>(0.44–1.15)<br>4	0.62<br>(0.36–0.77)<br>5
ABCB6	0.09<br>(0.08–0.12)<br>4	0.11<br>(0.10–0.12)<br>4	0.19<br>(0.14–0.23)<br>4	0.03<br>(0.03–0.03)<br>2	0.03<br>(0.02–0.03)<br>2
ABCB7	0.38<br>(0.28–0.50)<br>4	0.45<br>(0.40–0.51)<br>4	0.41<br>(0.30–0.54)<br>4	0.65<br>(0.55–0.77)<br>4	0.26<br>(0.10–0.39)<br>5
ABCB8	0.28<br>(0.22–0.36)<br>4	0.14<br>(0.10–0.19)<br>4	0.15<br>(0.12–0.18)<br>4	0.44<br>(0.17–0.60)<br>3	0.24<br>(0.20–0.34)<br>4
ABCB10	0.33<br>(0.25–0.43)<br>4	0.44<br>(0.38–0.56)<br>4	0.37<br>(0.22–0.56)<br>4	0.86<br>(0.43–1.01)<br>4	0.53<br>(0.50–0.56)<br>5
ABCC1	0.56<br>(0.44–0.76)<br>4	0.11<br>(0.09–0.13)<br>4	0.36<br>(0.20–0.46)<br>4	NQ	0.04<br>(0.01–0.12)<br>4
ABCC2	NQ	0.29<br>(0.16–0.55)<br>4	0.12<br>(0.07–0.20)<br>4	0.10<br>(0.06–0.14)<br>4	0.07<br>(0.03–0.13)<br>5
ABCC3	1.50<br>(1.16–1.78)<br>4	0.64<br>(0.52–0.72)<br>4	1.24<br>(1.06–1.43)<br>4	0.19<br>(0.10–0.29)<br>4	0.16<br>(0.13–0.22)<br>5
ABCC4	0.06<br>(0.03–0.11)<br>4	NQ	0.02<br>(0.02–0.03)<br>3	NQ	0.03<br>(0.03–0.03)<br>1
ABCC5	NQ	NQ	NQ	NQ	ND
ABCC6	ND	ND	NQ	0.04<br>(0.03–0.05)<br>2	0.03<br>(0.02–0.04)<br>3

ABCC10	NQ	NQ	NQ	ND	ND
ABCD1	0.09<br>(0.06–0.13)<br>4	0.14<br>(0.09–0.19)<br>4	0.12<br>(0.10–0.14)<br>4	2.46<br>(1.24–3.68)<br>4	1.02<br>(0.41–1.49)<br>5
ABCD3	4.85<br>(4.47–5.35)<br>4	4.61<br>(4.24–5.54)<br>4	4.18<br>(3.94–4.38)<br>4	5.39<br>(3.99–6.50)<br>4	3.08<br>(1.96–3.84)<br>5
ABCD4	0.02<br>(0.02–0.02)<br>2	0.08<br>(0.06–0.09)<br>4	0.05<br>(0.03–0.07)<br>3	0.07<br>(0.06–0.07)<br>2	0.06<br>(0.05–0.08)<br>4
ABCF1	1.64<br>(1.44–1.86)<br>4	1.17<br>(1.01–1.23)<br>4	1.33<br>(1.05–1.66)<br>4	1.92<br>(0.79–5.12)<br>4	0.67<br>(0.27–0.97)<br>5
ABCF2	0.54<br>(0.38–0.71)<br>4	0.13<br>(0.09–0.16)<br>4	0.30<br>(0.22–0.38)<br>4	0.07<br>(0.03–0.10)<br>4	0.10<br>(0.06–0.14)<br>4
ABCF3	0.28<br>(0.23–0.40)<br>4	0.35<br>(0.30–0.43)<br>4	0.30<br>(0.15–0.44)<br>4	0.56<br>(0.53–0.59)<br>2	0.35<br>(0.24–0.47)<br>3
ABCG2	0.01<br>(0.01–0.01)<br>1	0.44<br>(0.25–0.65)<br>4	0.58<br>(0.48–0.73)<br>4	0.61<br>(0.36–0.88)<br>4	0.55<br>(0.36–0.67)<br>4
ABCG5	ND	NQ	ND	0.04<br>(0.04–0.05)<br>2	0.04<br>(0.03–0.04)<br>2
ABCG8	ND	ND	ND	NQ	0.04<br>(0.04–0.04)<br>1

Table S8. Abundance of CYP enzymes in human 3D enteroids, primary enterocytes, and jejunal mucosa, Related to Figure 4. Protein levels (fmol/µg total protein) in different 3D enteroid formats cultured in ODM for 6 days (MBO, BO and AO) was compared to primary enterocyte and jejunal mucosa *in vivo* levels. Data from different donors (enteroids: Hu22-2jej, Hu22-3jej, Hu22-5jej, Hu18-9jej; enterocytes: Hu22-3jej, Hu22-5jej, Hu21-7jej, Hu21-14jej; jejunal mucosa: Hu22-2jej, Hu22-3jej, Hu22-5jej, Hu21-7jej, Hu21-14jej) are presented as mean (min – max) and n indicates the number of donors for which the protein was quantified. ND (not detected) indicates proteins that were not detected, and NQ (not quantified) refers to proteins that were identified but had expression levels below the limit of quantification.

Protein	MBO	AO	BO	Enterocytes	Mucosa
	Mean<br>(min - max)<br>n	Mean<br>(min - max)<br>n	Mean<br>(min - max)<br>n	Mean<br>(min - max)<br>n	Mean<br>(min - max)<br>n
CYP1A1	NQ	0.45<br>(0.29–0.63)<br>4	0.19<br>(0.13–0.34)<br>4	0.33<br>(0.08–0.58)<br>2	0.18<br>(0.06–0.30)<br>2
CYP2C9	ND	0.10<br>(0.05–0.15)<br>3	NQ	2.32<br>(0.84–3.64)<br>4	1.27<br>(1.08–1.47)<br>5
CYP2C18	1.00<br>(1.00–1.00)<br>1	2.64<br>(2.64–2.64)<br>1	1.64<br>(1.28–1.88)<br>4	3.06<br>(1.32–5.22)<br>4	1.16<br>(0.55–1.95)<br>5
CYP2C19	NQ	0.31<br>(0.07–0.49)<br>4	1.11<br>(0.34–1.49)<br>4	1.00<br>(0.79–1.11)<br>4	0.68<br>(0.35–0.97)<br>5
CYP2D6	ND	0.05<br>(0.04–0.05)<br>2	0.05<br>(0.05–0.05)<br>1	0.18<br>(0.18–0.18)<br>1	0.05<br>(0.05–0.05)<br>1
CYP2J2	0.08<br>(0.06–0.10)<br>2	NQ	NQ	0.39<br>(0.23–0.61)<br>4	0.25<br>(0.20–0.29)<br>3
CYP2S1	4.05<br>(3.30–4.91)<br>4	5.15<br>(2.16–7.15)<br>4	5.29<br>(2.39–7.46)<br>4	3.07<br>(0.89–4.99)<br>4	2.13<br>(1.94–2.39)<br>4
CYP2U1	0.05<br>(0.03–0.06)<br>3	NQ	NQ	NQ	NQ
CYP3A4	0.10<br>(0.06–0.21)<br>4	7.80<br>(5.83–9.72)<br>4	16.67<br>(5.85–32.83)<br>4	21.37<br>(18.38–26.96)<br>4	11.27<br>(9.28–16.40)<br>5
CYP3A5	0.08<br>(0.07–0.09)<br>2	1.17<br>(0.91–1.45)<br>4	2.16<br>(1.20–4.08)<br>4	2.58<br>(1.98–3.31)<br>4	1.20<br>(0.19–1.51)<br>5
CYP4F11	0.14<br>(0.09–0.25)<br>3	0.29<br>(0.09–0.57)<br>4	0.30<br>(0.25–0.36)<br>3	0.18<br>(0.16–0.21)<br>3	0.14<br>(0.11–0.17)<br>3
CYP4F12	0.14<br>(0.03–0.29)	0.41<br>(0.22–0.71)	0.65<br>(0.56–0.81)	0.50<br>(0.35–0.64)	0.22<br>(0.12–0.29)

	4	4	4	2	3
CYP4F2	0.41<br>(0.18–0.79)<br>4	1.43<br>(1.06–2.29)<br>4	2.36<br>(1.40–4.10)<br>4	4.21<br>(0.57–10.26)<br>4	2.65<br>(0.48–4.61)<br>5
CYP4V2	NQ	NQ	NQ	0.03<br>(0.02–0.03)<br>2	0.01<br>(0.01–0.02)<br>3
CYP4X1	NQ	NQ	NQ	ND	ND
CYP20A1	0.35<br>(0.29–0.44)<br>4	0.40<br>(0.32–0.52)<br>4	0.28<br>(0.24–0.32)<br>4	0.67<br>(0.50–0.89)<br>4	0.54<br>(0.41–0.78)<br>5
CYP27A1	0.06<br>(0.06–0.06)<br>3	2.44<br>(2.04–3.17)<br>4	0.61<br>(0.37–1.00)<br>4	7.35<br>(4.43–10.18)<br>4	4.35<br>(3.02–5.82)<br>5

Table S9. Abundance of other phase I enzymes (ADHs, ALDHs, CESs, FMOs) in human 3D enteroids, primary enterocytes, and jejunal mucosa, Related to Figure 4. Protein levels (fmol/µg total protein) in different 3D enteroid formats cultured in ODM for 6 days (MBO, AO and BO) was compared to primary enterocyte and jejunal mucosa *in vivo* levels. Data from different donors (enteroids: Hu22-2jej, Hu22-3jej, Hu22-5jej, Hu18-9jej; enterocytes: Hu22-3jej, Hu22-5jej, Hu21-7jej, Hu21-14jej; jejunal mucosa: Hu22-2jej, Hu22-3jej, Hu22-5jej, Hu21-7jej, Hu21-14jej) are presented as mean (min – max) and n indicates the number of donors for which the protein was quantified. ND (not detected) indicates proteins that were not detected, and NQ (not quantified) refers to proteins that were identified but had expression levels below the limit of quantification.

Protein	MBO	AO	BO	Enterocytes	Mucosa
	Mean<br>(min - max)<br>n	Mean<br>(min - max)<br>n	Mean<br>(min - max)<br>n	Mean<br>(min - max)<br>n	Mean<br>(min - max)<br>n
ADH1A	ND	ND	ND	NQ	NQ
ADH1B	NQ	NQ	NQ	0.34<br>(0.24–0.45)<br>4	9.54<br>(5.33–17.29)<br>5
ADH1C	0.14<br>(0.11–0.18)<br>3	26.69<br>(11.62–64.18)<br>4	21.83<br>(8.03–52.21)<br>4	49.31<br>(43.78–53.66)<br>4	53.55<br>(41.50–61.03)<br>5
ADH4	NQ	0.48<br>(0.11–1.12)<br>4	4.10<br>(3.19–5.16)<br>4	14.09<br>(9.89–22.31)<br>4	7.32<br>(4.61–10.93)<br>5
ADH5	9.16<br>(7.28–10.09)<br>4	13.82<br>(11.50–15.95)<br>4	11.36<br>(10.60–<br>12.21)<br>4	14.16<br>(9.52–26.60)<br>4	11.80<br>(6.82–18.49)<br>5
ADH6	0.05<br>(0.05–0.05)<br>1	0.33<br>(0.33–0.33)<br>1	0.42<br>(0.28–0.71)<br>4	6.41<br>(2.78–12.01)<br>4	3.83<br>(1.67–7.90)<br>5
ALDH16A1	1.07<br>(0.74–1.45)<br>4	0.75<br>(0.49–1.02)<br>4	1.08<br>(0.78–1.23)<br>4	1.22<br>(0.23–1.75)<br>3	1.41<br>(0.53–2.28)<br>5
ALDH18A1	6.62<br>(5.60–7.78)<br>4	11.76<br>(9.93–13.42)<br>4	11.52<br>(10.00–<br>12.49)<br>4	8.48<br>(6.38–9.86)<br>4	4.96<br>(3.78–5.85)<br>5
ALDH1A1	24.31<br>(21.46–30.81)<br>4	226.09<br>(158.79–<br>295.09)<br>4	90.31<br>(82.66–<br>95.19)<br>4	83.20<br>(70.72–<br>104.30)<br>4	80.50<br>(65.29–106.34)<br>5
ALDH1A3	3.87<br>(1.79–7.53)<br>4	7.82<br>(5.51–11.12)<br>4	2.39<br>(1.41–4.27)<br>4	NQ	1.36<br>(1.03–1.87)<br>5
ALDH1B1	6.46<br>(6.29–6.82)<br>4	3.76<br>(3.18–4.56)<br>4	9.11<br>(6.04–14.53)<br>4	2.40<br>(1.29–3.39)<br>4	3.73<br>(3.25–4.22)<br>5
ALDH1L1	0.38<br>(0.08–0.91)<br>3	0.64<br>(0.06–1.70)<br>3	1.68<br>(0.05–4.76)<br>4	0.03<br>(0.03–0.03)<br>1	0.07<br>(0.04–0.11)<br>3

ALDH1L2	ND	ND	ND	ND	0.04 (0.04–0.04) 1
ALDH2	25.62 (22.79–28.70) 4	38.79 (33.24–46.40) 4	39.52 (36.43–42.96) 4	36.47 (32.85–39.16) 4	25.01 (22.97–28.90) 5
ALDH3A1	9.47 (8.48–11.64) 4	3.90 (3.49–4.34) 4	3.62 (3.02–4.07) 4	0.26 (0.26–0.27) 2	0.12 (0.06–0.22) 4
ALDH3A2	9.54 (7.67–10.86) 4	10.79 (7.32–13.75) 4	7.68 (6.89–9.35) 4	10.19 (9.27–11.68) 4	6.65 (3.04–9.69) 5
ALDH3B1	0.03 (0.03–0.03) 1	0.22 (0.15–0.33) 4	0.09 (0.08–0.11) 3	0.11 (0.08–0.13) 2	0.07 (0.06–0.10) 3
ALDH4A1	1.43 (1.12–1.80) 4	0.81 (0.65–0.97) 4	0.65 (0.38–0.92) 4	NQ	0.10 (0.05–0.18) 3
ALDH5A1	0.37 (0.31–0.46) 4	0.85 (0.64–1.00) 4	0.22 (0.20–0.26) 4	3.55 (2.21–4.43) 4	2.29 (1.10–3.42) 5
ALDH6A1	0.45 (0.41–0.48) 4	1.34 (1.22–1.57) 4	0.61 (0.48–0.77) 4	14.84 (9.60–18.75) 4	6.41 (3.66–10.42) 5
ALDH7A1	9.21 (6.63–12.51) 4	7.05 (4.69–9.71) 4	6.60 (4.94–8.73) 4	6.21 (4.96–7.09) 4	4.62 (3.99–6.05) 5
ALDH9A1	5.16 (4.85–5.55) 4	7.79 (6.42–8.74) 4	4.73 (4.36–5.06) 4	11.53 (8.44–17.46) 4	8.49 (4.60–10.29) 5
CES1	0.75 (0.28–1.38) 3	1.24 (0.59–2.45) 3	2.77 (0.09–5.09) 4	0.64 (0.47–0.78) 4	1.37 (1.05–1.80) 5
CES2	5.51 (3.53–6.41) 4	55.34 (50.52–59.37) 4	28.74 (22.37–32.56) 4	54.40 (43.63–60.23) 4	37.14 (26.32–50.67) 5
CES3	0.28 (0.18–0.48) 4	0.24 (0.19–0.33) 4	0.17 (0.06–0.31) 4	0.35 (0.16–0.74) 4	0.39 (0.16–0.62) 4
FMO1	NQ	NQ	NQ	0.40 (0.09–1.20) 4	0.41 (0.08–1.48) 5
FMO4	NQ	0.04 (0.03–0.05) 2	0.01 (0.01–0.01) 1	0.24 (0.14–0.31) 4	0.07 (0.04–0.10) 3
FMO5	NQ	0.60 (0.44–0.74) 4	0.41 (0.35–0.45) 4	1.10 (0.93–1.27) 4	0.40 (0.08–0.55) 5

Table S10. Abundance of UDP-glucuronosyltransferases (UGTs) in human 3D enteroids, primary enterocytes, and jejunal mucosa, Related to Figure 4. Protein levels (fmol/µg total protein) in different 3D enteroid formats cultured in ODM for 6 days (MBO, AO and BO) was compared to primary enterocyte and jejunal mucosa *in vivo* levels. Data from different donors (enteroids: Hu22-2jej, Hu22-3jej, Hu22-5jej, Hu18-9jej; enterocytes: Hu22-3jej, Hu22-5jej, Hu21-7jej, Hu21-14jej; jejunal mucosa: Hu22-2jej, Hu22-3jej, Hu22-5jej, Hu21-7jej, Hu21-14jej) are presented as mean (min – max) and n indicates the number of donors for which the protein was quantified. ND (not detected) indicates proteins that were not detected, and NQ (not quantified) refers to proteins that were identified but had expression levels below the limit of quantification.

Protein	MBO	AO	BO	Enterocytes	Mucosa
	Mean<br>(min - max)<br>n	Mean<br>(min – max)<br>n	Mean<br>(min - max)<br>n	Mean<br>(min - max)<br>n	Mean<br>(min - max)<br>n
UGT1A1	0.91<br>(0.47–1.96)<br>4	5.98<br>(2.88–8.78)<br>4	5.34<br>(5.01–5.98)<br>4	5.65<br>(5.30–6.20)<br>3	2.43<br>(1.09–4.00)<br>5
UGT1A3	ND	NQ	NQ	0.17<br>(0.15–0.19)<br>2	0.14<br>(0.14–0.14)<br>1
UGT1A4	ND	0.16<br>(0.05–0.42)<br>4	0.51<br>(0.05–1.38)<br>3	0.14<br>(0.12–0.15)<br>2	0.07<br>(0.04–0.10)<br>2
UGT1A5	ND	0.11<br>(0.10–0.11)<br>2	0.17<br>(0.03–0.32)<br>2	NQ	0.10<br>(0.10–0.10)<br>1
UGT1A6	4.04<br>(2.71–6.14)<br>4	18.88<br>(14.80–25.27)<br>4	15.09<br>(11.00–19.60)<br>4	7.97<br>(6.29–10.65)<br>4	5.55<br>(3.84–7.86)<br>5
UGT1A8	ND	0.22<br>(0.14–0.42)<br>4	0.23<br>(0.13–0.41)<br>4	0.06<br>(0.06–0.06)<br>1	NQ
UGT1A10	0.72<br>(0.29–1.06)<br>4	6.43<br>(5.93–7.49)<br>4	5.44<br>(4.46–7.51)<br>4	2.73<br>(2.35–3.31)<br>3	1.80<br>(1.25–2.75)<br>4
UGT2A3	0.25<br>(0.04–0.58)<br>4	1.85<br>(0.79–3.80)<br>4	0.34<br>(0.10–0.51)<br>4	8.50<br>(2.37–14.16)<br>4	6.17<br>(4.25–8.06)<br>5
UGT2B7	NQ	0.50<br>(0.06–1.11)<br>4	0.07<br>(0.02–0.11)<br>4	5.92<br>(3.61–8.43)<br>4	2.69<br>(1.36–3.77)<br>5
UGT2B17	0.10<br>(0.08–0.11)<br>3	16.16<br>(0.51–24.25)<br>4	10.54<br>(3.60–19.08)<br>3	38.87<br>(0.41–71.89)<br>4	18.42<br>(12.68–29.94)<br>4

Table S11. Abundance of sulfotransferases (SULTs) in human 3D enteroids, primary enterocytes, and jejunal mucosa, Related to Figure 4. Protein levels (fmol/µg total protein) in different 3D enteroid formats cultured in ODM for 6 days (MBO, BO and AO) was compared to primary enterocyte and jejunal mucosa *in vivo* levels. Data from different donors (enteroids: Hu22-2jej, Hu22-3jej, Hu22-5jej, Hu18-9jej; enterocytes: Hu22-3jej, Hu22-5jej, Hu21-7jej, Hu21-14jej; jejunal mucosa: Hu22-2jej, Hu22-3jej, Hu22-5jej, Hu21-7jej, Hu21-14jej) are presented as mean (min – max) and n indicates the number of donors for which the protein was quantified. ND (not detected) indicates proteins that were not detected, and NQ (not quantified) refers to proteins that were identified but had expression levels below the limit of quantification.

Protein	MBO	AO	BO	Enterocytes	Mucosa
	Mean<br>(min - max)<br>n	Mean<br>(min - max)<br>n	Mean<br>(min - max)<br>n	Mean<br>(min - max)<br>n	Mean<br>(min - max)<br>n
SULT1A1	2.07<br>(0.77–3.36)<br>4	14.65<br>(13.61–15.55)<br>4	12.11<br>(7.82–19.08)<br>4	41.63<br>(28.55–56.86)<br>4	27.10<br>(19.33–39.35)<br>5
SULT1A2	ND	0.33<br>(0.13–0.54)<br>2	0.57<br>(0.25–0.88)<br>2	1.29<br>(0.70–2.27)<br>4	1.01<br>(0.73–1.18)<br>3
SULT1A3	5.13<br>(4.60–5.68)<br>4	33.41<br>(23.12–44.50)<br>4	19.87<br>(15.60–25.74)<br>4	81.66<br>(70.74–91.34)<br>4	81.77<br>(62.99–90.89)<br>5
SULT1B1	NQ	1.47<br>(0.25–2.49)<br>4	1.10<br>(0.68–2.20)<br>4	35.97<br>(27.35–43.03)<br>4	23.85<br>(9.63–36.42)<br>5
SULT1C2	NQ	0.26<br>(0.18–0.38)<br>3	0.83<br>(0.08–1.67)<br>4	0.75<br>(0.11–1.73)<br>3	0.54<br>(0.14–1.05)<br>3
SULT1E1	ND	ND	NQ	0.94<br>(0.63–1.56)<br>4	1.15<br>(0.68–1.82)<br>5
SULT2A1	0.06<br>(0.03–0.10)<br>3	11.71<br>(7.65–16.69)<br>4	1.37<br>(0.52–2.47)<br>4	26.29<br>(16.56–33.19)<br>4	17.11<br>(9.79–27.23)<br>5

Table S12. Abundance of other phase II enzymes (GSTs and NATs) in human 3D enteroids, primary enterocytes, and jejunal mucosa, Related to Figure 4. Protein levels (fmol/µg total protein) in different 3D enteroid formats cultured in ODM for 6 days (MBO, BO and AO) was compared to primary enterocyte and jejunal mucosa *in vivo* levels. Data from different donors (enteroids: Hu22-2jej, Hu22-3jej, Hu22-5jej, Hu18-9jej; enterocytes: Hu22-3jej, Hu22-5jej, Hu21-7jej, Hu21-14jej; jejunal mucosa: Hu22-2jej, Hu22-3jej, Hu22-5jej, Hu21-7jej, Hu21-14jej) are presented as mean (min – max) and n indicates the number of donors for which the protein was quantified. ND (not detected) indicates proteins that were not detected, and NQ (not quantified) refers to proteins that were identified but had expression levels below the limit of quantification.

Protein	MBO	AO	BO	Enterocytes	Mucosa
	Mean<br>(min - max)<br>n	Mean<br>(min - max)<br>n	Mean<br>(min - max)<br>n	Mean<br>(min - max)<br>n	Mean<br>(min - max)<br>n
GSTA1	0.54<br>(0.03–1.01)<br>4	113.32<br>(46.33–179.61)<br>4	39.50<br>(30.81–45.45)<br>4	133.79<br>(116.35–167.28)<br>4	134.76<br>(34.27–193.65)<br>5
GSTA2	ND	NQ	NQ	NQ	NQ
GSTCD	0.08<br>(0.08–0.09)<br>4	0.04<br>(0.02–0.05)<br>3	0.05<br>(0.02–0.07)<br>4	NQ	NQ
GSTK1	7.17<br>(6.52–7.65)<br>4	14.28<br>(12.41–17.12)<br>4	12.35<br>(11.20–14.01)<br>4	39.12<br>(36.21–41.05)<br>4	18.68<br>(11.51–21.87)<br>5
GSTM1	2.29<br>(2.16–2.43)<br>2	3.12<br>(2.72–3.52)<br>2	2.44<br>(2.32–2.55)<br>2	2.45<br>(2.00–2.90)<br>2	3.05<br>(2.71–3.43)<br>3
GSTM2	0.90<br>(0.47–1.28)<br>4	0.62<br>(0.41–1.13)<br>4	0.23<br>(0.16–0.28)<br>4	0.24<br>(0.18–0.37)<br>3	2.80<br>(1.36–5.24)<br>5
GSTM3	11.81<br>(7.53–19.72)<br>4	8.27<br>(5.36–16.13)<br>4	10.16<br>(6.00–19.46)<br>4	4.54<br>(1.43–7.88)<br>4	8.58<br>(2.78–16.21)<br>5
GSTM4	8.35<br>(3.10–18.32)<br>4	6.79<br>(1.05–14.04)<br>4	7.48<br>(3.16–12.05)<br>4	3.73<br>(0.42–8.38)<br>4	9.86<br>(1.96–22.44)<br>5
GSTO1	13.12<br>(8.19–16.23)<br>4	24.55<br>(16.45–29.83)<br>4	17.40<br>(13.06–20.52)<br>4	30.85<br>(23.73–44.54)<br>4	26.95<br>(13.43–38.11)<br>5
GSTP1	120.29<br>(104.54–136.60)<br>4	181.65<br>(145.80–217.55)<br>4	124.46<br>(105.61–150.17)<br>4	34.91<br>(14.48–46.19)<br>4	61.50<br>(51.58–82.56)<br>5
GSTT1	1.93<br>(1.78–2.08)<br>3	2.85<br>(2.63–3.06)<br>3	1.97<br>(1.34–2.77)<br>3	3.52<br>(2.21–4.97)<br>3	4.39<br>(2.29–8.64)<br>4

GSTT2B	0.51<br>(0.51–0.51)<br>1	0.25<br>(0.25–0.25)<br>1	0.23<br>(0.23–0.23)<br>1	ND	0.12<br>(0.05–0.19)<br>2
GSTZ1	2.33<br>(1.65–3.63)<br>4	1.61<br>(1.21–2.22)<br>4	1.33<br>(1.02–1.84)<br>4	1.91<br>(1.61–2.34)<br>3	1.28<br>(1.07–1.58)<br>3
NAT1	0.36<br>(0.29–0.43)<br>4	1.71<br>(1.38–2.56)<br>4	1.14<br>(0.75–1.56)<br>4	0.99<br>(0.39–1.63)<br>4	1.01<br>(0.29–2.06)<br>5
NAT2	0.56<br>(0.56–0.56)<br>1	0.67<br>(0.45–0.94)<br>3	0.57<br>(0.45–0.70)<br>3	0.31<br>(0.31–0.31)<br>1	1.56<br>(0.60–2.53)<br>2
NAT10	1.24<br>(1.09–1.41)<br>4	0.29<br>(0.22–0.36)<br>4	0.86<br>(0.55–1.12)<br>4	0.12<br>(0.07–0.21)<br>3	0.20<br>(0.07–0.29)<br>5
NAT14	NQ	NQ	ND	ND	ND

Table S13. Input parameters for the terfenadine PBPK model, Related to Figure 8.

Parameter	Value	Source
MW	471.7	Pubchem
Log P	5.01	ter Laak *et. al* 1994[S5]
pKa	8.6 (base)	
BP	0.78	Predicted in the simcyp simulator
fu_{plasma}	0.03	Chen *et. al* 2007[S6]
Absorption	ADAM model (solution)	Jamei *et. al* 2009[S7]
Fu_{gut}	0.15	estimated
Peff,man (10^{-4} cm/s)	13.51	Predicted using the mechanistic permeability model[S8]
Vss (L/kg)	27.5	
Elimination – HLM (ml/min/mg microsomal protein)	13 342	CYP3A4
Elimination – HLM (ml/min/mg microsomal protein)	272	Non-CYP3A4 metabolism

References

S1. Wandel, C., Witte, J.S., Hall, J.M., Stein, C.M., Wood, A.J.J., and Wilkinson, G.R. (2000). CYP3A activity in African American and European American men: Population differences and functional effect of the CYP3A4*1B 5'-promoter region polymorphism. Clin. Pharmacol. Ther. *68*, 82–91. https://doi.org/10.1067/mcp.2000.108506.

S2. Stoch, S.A., Friedman, E., Maes, A., Yee, K., Xu, Y., Larson, P., Fitzgerald, M., Chodakewitz, J., and Wagner, J.A. (2009). Effect of different durations of ketoconazole dosing on the single-dose pharmacokinetics of midazolam: Shortening the paradigm. J. Clin. Pharmacol. *49*, 398–406. https://doi.org/10.1177/0091270008331133.

S3. Templeton, I., Peng, C.C., Thummel, K.E., Davis, C., Kunze, K.L., and Isoherranen, N. (2010). Accurate prediction of dose-dependent CYP3A4 inhibition by itraconazole and its metabolites from in vitro inhibition data. Clin. Pharmacol. Ther. *88*, 499–505. https://doi.org/10.1038/clpt.2010.119.

S4. Krishna, G., Ma, L., Prasad, P., Moton, A., Martinho, M., and O'Mara, E. (2012). Effect of posaconazole on the pharmacokinetics of simvastatin and midazolam in healthy volunteers. Expert Opin. Drug Metab. Toxicol. *8*, 1–10. https://doi.org/10.1517/17425255.2012.639360.

S5. Ter Laak, A.M., Tsai, R.S., Donné-Op Den Kelder, G.M., Carrupt, P.-A., Testa, B., and Timmerman, H. (1994). Lipophilicity and hydrogen-bonding capacity of H1-antihistaminic agents in relation to their central sedative side-effects. Eur. J. Pharm. Sci. *2*, 373–384. https://doi.org/10.1016/0928-0987(94)00065-4.

S6. Chen, C. (2007). Some pharmacokinetic aspects of the lipophilic terfenadine and zwitterionic fexofenadine in humans. Drugs R. D. *8*, 301–314. https://doi.org/10.2165/00126839-200708050-00004.

S7. Jamei, M., Turner, D., Yang, J., Neuhoff, S., Polak, S., Rostami-Hodjegan, A., and Tucker, G. (2009). Population-based mechanistic prediction of oral drug absorption. AAPS J. *11*, 225–237. https://doi.org/10.1208/s12248-009-9099-y.

S8. Pade, D., Jamei, M., Rostami-Hodjegan, A., and Turner, D.B. (2017). Application of the MechPeff model to predict passive effective intestinal permeability in the different regions of the rodent small intestine and colon. Biopharm. Drug Dispos. *38*, 94–114. https://doi.org/10.1002/bdd.2072.
